# Virtual Reality–Based Cue Exposure and Aversion Therapy for Alcohol Dependence: A Randomized Controlled Trial

**DOI:** 10.1111/adb.70166

**Published:** 2026-05-06

**Authors:** Haoyu Zhao, Xiaotong Ying, Xiaoyu Du, Genhui Ren, Hongdu Deng, Wenhui Li, Jiali Wang, Ming Chen, Zihang Shao, Jingshu Zhang, Lanci Liu, Jie Zhang, Ping Cui, Chunyan Li, Xinyou Wang, Ying Xu, Junjun Zhang, Dan Wang, Chuansheng Wang

**Affiliations:** ^1^ The Second Affiliated Hospital of Henan Medical University Xinxiang Henan China

**Keywords:** alcohol dependence, attentional bias, aversion therapy, cue exposure therapy, virtual reality

## Abstract

Alcohol dependence (ad) is characterized by a high relapse rate. Virtual reality (VR) technology can provide immersive cue exposure therapy (VR‐CET) and aversion therapy (VR‐AT). This study aimed to evaluate the efficacy of VR‐CET, VR‐AT and their combination on craving, emotional and sleep states, attentional bias and relapse rate in patients with ad. In this single‐centre randomized controlled trial, male inpatients with ad were randomly assigned to one of four groups: control, VR‐CET, VR‐AT or combined VR‐CET + AT (target *n* = 25 per group; 80 completed, 20 per group). The interventions spanned 15 days with eight sessions (VR‐CET + AT ~20 min/session; others ~10 min). Assessments were conducted before and after treatment using the Visual Analogue Scale (VAS), the Pennsylvania Alcohol Craving Scale (PACS), the Hamilton Depression Scale (HAMD), the Hamilton Anxiety Scale (HAMA) and the Pittsburgh Sleep Quality Index (PSQI). Eye‐tracking and grasping indices in VR environments were used to assess attentional bias (e.g., alcohol‐cue fixation time ratio). Relapse was evaluated by telephone at 4 and 12 weeks post‐treatment. Statistical analyses used Shapiro–Wilk tests and ANOVA/Kruskal–Wallis tests with appropriate post hoc comparisons (*α* = 0.05). All groups showed significant pre‐ to post‐treatment improvements in PACS, HAMD, HAMA, PSQI and VAS scores (all *p* < 0.001). Between‐group comparisons at post‐treatment revealed significant differences in alcohol‐cue fixation time ratio (*p* < 0.05), with the VR‐CET + AT group showing a lower fixation time ratio than the control group. VAS scores also differed among groups (*p* < 0.05), with the control group showing higher values than the VR‐CET + AT group. Changes in alcohol‐cue fixation time ratio from pre‐ to post‐treatment were significantly greater in the VR‐CET + AT group than in the control group. Relapse rates at 4 and 12 weeks (47/80 reached by telephone follow‐up) did not significantly differ among groups (both *p* > 0.05). Combining VR‐CET with VR‐AT reduced craving (VAS) and attentional bias (alcohol‐cue fixation time ratio) beyond standard care, whereas all groups improved on clinical scales. Larger and longer trials are warranted to further clarify relapse outcomes.

**Trial Registration:** Chinese Clinical Trial Registry, ChiCTR2500110026. Registered 29 September 2025 (retrospectively registered)

## Graphical abstract

Virtual reality–based cue exposure therapy and aversion therapy were evaluated in male inpatients with alcohol dependence. The combined VR‐CET+AT intervention reduced craving and alcohol‐related attentional bias beyond standard care, whereas relapse rates did not differ significantly at 4 or 12 weeks. These findings support VR‐based interventions as promising adjunctive approaches for alcohol dependence.

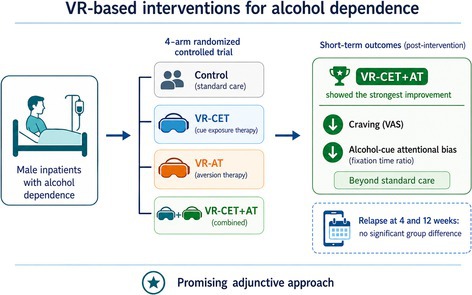

## 1 Introduction

Alcohol dependence (ad) is a pattern of alcohol use that causes clinically significant impairment or distress [1], characterized by compulsive drinking and a loss of control over alcohol consumption [2]. ad is a major global public health issue, with one in eight adults in the United States reporting high‐risk drinking behaviours in the past year and a high lifetime prevalence of ad. In the United Kingdom, the prevalence of heavy drinking and ad is also notably high [3]. In recent years, alcohol consumption and alcohol use disorder have shown an upward trend in China, accompanied by an increasing burden of alcohol‐related health problems. Large‐scale epidemiological studies and national surveys have reported rising per capita alcohol consumption and a growing prevalence of alcohol‐related disorders in the Chinese population [4]. Chronic AD not only severely affects physical health, leading to conditions such as liver disease, gastrointestinal issues, cardiovascular diseases and immune system impairment [5], but also has negative impacts on families and society [6]. These issues worsen as the frequency of relapse increases [7].

Psychological craving is one of the primary reasons for relapse. A substantial body of neurobiological and social‐psychological research suggests that reducing cravings is crucial for maintaining alcohol abstinence and preventing relapse [8]. Currently, pharmacological treatments such as naltrexone and acamprosate are commonly used to address psychological cravings in ad patients [9]. However, the efficacy of these treatments is limited, and they inevitably cause adverse side effects, including liver damage, diarrhoea, headache and dizziness, which in turn reduce patient adherence to treatment [10].

In parallel with pharmacotherapy, several psychological and behavioural interventions have been developed to specifically target craving and cue reactivity. For example, cue exposure therapy (CET) seeks to attenuate cue‐induced craving through repeated exposure to alcohol‐related stimuli without consumption (i.e., extinction learning). Cognitive behavioural therapy, by contrast, aims to enhance coping skills and reduce automatic responses to alcohol cues [11, 12]. Nonetheless, the translation of these approaches into real‐world settings can be challenging because traditional cue presentations (e.g., pictures or videos) often lack ecological validity.

Virtual reality (VR) offers a promising way to deliver cue exposure in immersive, realistic drinking contexts, and VR‐based CET (VR‐CET) has shown feasibility and potential benefits for reducing craving and attentional bias in alcohol use disorder [13]. A recent quantitative meta‐analysis by Kiyak et al. [14] provided systematic evidence supporting the efficacy of cue exposure–based interventions in reducing craving in alcohol use disorder, although effects on long‐term drinking outcomes remain heterogeneous. In addition, a systematic review by Durl et al. [15] summarized the use of VR technologies in alcohol‐related research, highlighting the potential of immersive environments to enhance ecological validity in both assessment and intervention. By contrast, aversion therapy (AT) aims to establish or strengthen negative associations with alcohol‐related stimuli, but conventional AT approaches (e.g., emetic or electrical methods) are limited by safety and acceptability concerns [16, 17]. Integrating AT into VR may provide an immersive yet safer and more acceptable way to deliver aversive learning. To our knowledge, however, randomized controlled trials that combine VR‐CET with VR‐based AT (VR‐AT) within a single intervention framework remain scarce. Therefore, we conducted a randomized controlled trial to evaluate the effects of VR‐CET, VR‐AT and their combination on craving, emotional and sleep states, attentional bias and relapse outcomes in patients with ad.

### 1.1 VR Technology

VR technology is a computer system that can create and enable users to experience virtual environments, designed to simulate reality and create an immersive, interactive world. When users perceive that the environment accurately simulates the real‐world experience they aim to replicate, they can fully immerse themselves in VR [18]. In recent years, the commercial application costs of VR technology have significantly decreased, and VR systems are no longer expensive; computers and smartphones can now enable VR functionality, making VR technology more widely accessible. As an intervention method that offers flexible treatment time, low cost and personalized options, VR technology has increasingly been applied in the field of mental disorders, especially in the treatment of anxiety disorders, post‐traumatic stress disorder and depression, with promising progress [19]. In the field of addiction, previous studies have suggested that VR‐assisted therapy for ad may have beneficial effects [20, 21, 22, 23, 24, 25]. For example, Pennington et al. conducted a study in which veterans with traumatic brain injuries and alcohol use disorders navigated a virtual outdoor environment on a stationary bike to complete tasks, enhancing their self‐control over alcohol and reducing relapse [24].

### 1.2 CET

CET is a behavioural intervention for substance use disorders that involves exposing patients to addiction‐related cues in order to reduce their impulsive urges to use substances [26]. CET can be used to explore alcohol craving responses by presenting alcohol‐related cues to individuals in a non‐realistic experimental or clinical setting, thereby inducing psychological and physiological cravings for alcohol in a controlled environment [27, 28]. The goal of CET is to extinguish drinking urges through repeated exposure [29]. Specifically, patients are systematically and repeatedly exposed to alcohol‐related stimuli without engaging in actual drinking behaviour, which theoretically reduces their psychological and physiological responses to alcohol‐related cues and extinguishes the initial conditioned reflexes (e.g., craving) associated with alcohol cues [30].

### 1.3 AT

AT is a treatment approach that aims to eliminate maladaptive behaviours through punitive mechanisms. The core principle of AT is to immediately administer an aversive stimulus (e.g., mild electric shock, acupuncture or emetic agents) when the maladaptive behaviour is about to occur or is in progress, thereby inducing a strong sense of aversion [31]. Common forms of AT include electro‐AT and pharmacological AT. Although these conventional methods can reduce patients' craving for alcohol, they are often associated with considerable discomfort and low safety. Integrating AT with VR technology may effectively overcome these limitations.

### 1.4 Eye‐Tracking Technology

Eye‐tracking technology is a method used to measure eye position and ocular movements, primarily aimed at understanding how observers direct their gaze and shift attention when viewing different objects or scenes. Attention bias (AB) refers to the preferential allocation or avoidance of attention toward certain stimuli during information processing. In the field of substance addiction, AB is defined as the tendency of substance‐related cues to preferentially capture the attention of individuals with substance dependence [32]. It is considered one of the core features of addiction and is closely associated with the maintenance of abstinence as well as relapse [33]. In recent years, eye‐tracking technology has increasingly been employed as a tool to examine gaze direction, providing an accurate assessment of AB and serving as an indicator of craving [34]. Compared with flat‐screen displays, the immersive environment provided by VR is more advantageous for demonstrating AB in patients. Combining VR technology with eye‐tracking technology may therefore offer a more effective means of reflecting patients' craving.

### 1.5 Motion Capture Technology

Motion capture technology records the movement trajectories of the human body or objects and converts them into digital data, which can then be explored and analysed for applications across various fields. In the context of substance addiction, integrating motion capture technology with VR enables the assessment of patients' reaching or grasping actions toward alcohol‐related cues. Such actions can reflect the approach tendencies of patients toward these cues and thereby serve as an indicator of craving.

### 1.6 Objectives of the Study

AD is characterized by high prevalence, disability and relapse rates, all of which have shown an increasing trend in recent years. The persistently high relapse rate imposes substantial negative impacts on patients, families and society. Reducing craving is considered the key to lowering relapse; however, effective interventions specifically targeting craving remain limited. This study evaluates the efficacy of three therapeutic approaches—VR‐CET, VR‐AT and their combination (VR‐CET + AT)—to explore whether these interventions may help reduce psychological craving and related relapse risk in patients with ad, and to examine their potential as a promising VR‐based adjunctive intervention model for ad.

## 2 Methods

### 2.1 Design and Construction of VR Scenarios

Three types of VR scenarios were used in this study. The aversion condition consisted of 360° first‐person videos depicting negative alcohol‐related consequences in realistic Chinese contexts. The cue‐exposure condition was an interactive virtual restaurant environment containing both alcohol‐related and non‐alcohol‐related objects, allowing free observation and grasping while eye‐tracking and grasping data were recorded. The control condition used neutral, non‐alcohol‐related relaxation videos showing calming natural scenes. All scenarios were delivered using the HTC VIVE Focus 3 platform. Preliminary testing indicated that the aversion and cue‐exposure scenarios were realistic and capable of eliciting the intended responses, whereas the relaxation videos were acceptable and relaxing to patients. Detailed scenario development, construction and validation procedures are provided in the Supporting Information.

### 2.2 Participants

#### 2.2.1 Sample Size

Sample size estimation was performed using G*Power Version 3.1.9.7. Based on a one‐way analysis of variance (ANOVA) with four groups (fixed effects, omnibus test), a two‐sided significance level of *α* = 0.05 and a power of 0.80 were specified, assuming a medium effect size of Cohen's *f* = 0.25. This effect size was consistent with that reported in a randomized controlled trial comparing VR CET with standard treatment in patients with AD [13]. The sample size calculation was based on the primary outcome of the study, namely, craving intensity assessed by the Visual Analogue Scale (VAS). The calculation indicated that a total sample size of 100 participants would be required, with 25 participants in each group.

#### 2.2.2 Recruit Participants

From September 2024 to May 2025, a total of 101 male inpatients with AD were recruited from the Addiction Medicine Department of the Second Affiliated Hospital of Henan Medical University. After enrollment and confirmation of eligibility, participants were allocated in an approximately 1:1:1:1 ratio to the control, VR‐CET, VR‐AT or VR‐CET + AT group using a random number table. The allocation sequence was generated by a researcher who was not involved in outcome assessment, and group assignment was implemented after participant enrollment. This process resulted in 25 participants in the control group, 26 in the VR‐CET group, 25 in the VR‐AT group and 25 in the VR‐CET + AT group.

Inclusion criteria: (1) meeting the ICD‐10 diagnostic criteria for AD; (2) male sex, age 18–60 years; (3) primary school education or above; (4) no significant somatic withdrawal symptoms (Clinical Institute Withdrawal Assessment for Alcohol score < 7); and (5) ability to cooperate with VR treatment, willingness to complete follow‐up assessments and provision of written informed consent.

Exclusion criteria: (1) severe physical illness or a history of organic brain disease; (2) a history of substance abuse other than alcohol and nicotine; (3) a history of other psychiatric disorders; and (4) visual impairment that would interfere with viewing the VR environment.

Withdrawal criteria: (1) withdrawal of informed consent; (2) adverse events or clinical deterioration such that continued participation would not be in the patient's best interest; (3) completion of ≤ 4 of the 8 intervention sessions, as insufficient exposure was unlikely to produce meaningful therapeutic effects and could bias efficacy estimates.

The study protocol was approved by the Ethics Committee of the Second Affiliated Hospital of Henan Medical University, and all participants were fully informed of the study objectives and provided written informed consent.

### 2.3 Research Technique

#### 2.3.1 Measurement Tools

Basic information: Demographic data were collected, including age, sex, educational level, religious belief, marital status, spousal relationship, type of household registration, occupation, economic income, history of chronic diseases, smoking status, smoking duration and number of cigarettes smoked per day. These variables were collected as part of routine baseline clinical assessment to characterize participants' physical status and to provide a comprehensive description of baseline characteristics. Information on alcohol use was also collected, including total years of drinking, type of alcoholic beverage, drinking frequency and daily alcohol intake.

Clinical Institute Withdrawal Assessment for Alcohol‐Revised (CIWA‐Ar) [35]: This scale consists of 10 items, including nausea and vomiting, tremor, sweating, tactile disturbances, auditory disturbances, visual disturbances, anxiety, agitation, headache or fullness in the head and orientation. Each item is scored, with a total score of 0–9 indicating very mild withdrawal, 10–15 mild withdrawal, 16–20 moderate withdrawal and 21–67 severe withdrawal. The CIWA‐Ar is used to assess the severity of alcohol withdrawal symptoms.

VAS [36]: Participants were asked to indicate the intensity of their craving on a 10‐cm horizontal line, with ‘0’ on the far left representing no craving and ‘10’ on the far right representing the strongest craving.

Pennsylvania Alcohol Craving Scale (PACS) [37]: This is a self‐report measure that assesses the frequency, intensity, and duration of alcohol craving, the ability to resist drinking, and the overall level of previous alcohol craving. Each item is scored from 0 to 6. The scale is used to evaluate the severity of patients' alcohol craving.

Hamilton Anxiety Scale (HAMA) [38]: This scale primarily assesses somatic and psychological anxiety. A score greater than 7 indicates the presence of anxiety symptoms, with higher scores reflecting more severe anxiety. It can be used to evaluate both the severity of anxiety and the effectiveness of various interventions.

Hamilton Depression Scale (HAMD) [39]: This scale primarily assesses anxiety‐somatization, weight, cognitive impairment, retardation and sleep disturbance. A score greater than 8 indicates the presence of depressive symptoms, with higher scores reflecting greater severity. The total score provides a reliable measure of the overall severity of depressive symptoms.

Pittsburgh Sleep Quality Index (PSQI) [40]: This is a self‐report questionnaire used to assess sleep quality over the past month.

Relapse status: Assessed by standardized telephone follow‐up at 4 and 12 weeks after completion of the intervention. For the purpose of this study, relapse was operationally defined as any alcohol consumption, regardless of amount or frequency, occurring between the end of the intervention and the relevant follow‐up time point. Participants who reported no alcohol use during that interval were classified as abstinent. All follow‐up interviews were conducted by a single trained clinical psychiatrist who was not involved in group allocation or intervention delivery. At each follow‐up assessment, participants were asked whether they had consumed any alcohol since the end of the intervention, and responses were recorded using the same predefined procedure at both time points. Participants who could not be contacted were classified as lost to follow‐up.

Eye‐tracking data: During VR‐CET treatment, eye‐tracking data were recorded by the alcohol craving assessment system. The variables included ‘time fixating on alcohol‐related cues’, ‘number of alcohol‐related fixations’, ‘time fixating on non‐alcohol‐related cues’ and ‘number of non‐alcohol‐related fixations’.

Grasping data: During VR‐CET treatment, grasping data were recorded by the alcohol craving assessment system. The variables included ‘time to grasp alcohol‐related cues’, ‘number of alcohol‐related grasps’, ‘time to grasp non‐alcohol‐related cues’ and ‘number of non‐alcohol‐related grasps’.

The questionnaire‐based assessment instruments used in this study are well‐established measures with demonstrated reliability and validity in clinical and research settings. Craving intensity was assessed using the VAS, which has been widely applied in AD research. The PACS is a validated instrument for evaluating the severity of alcohol craving. Symptoms of anxiety and depression were assessed using the HAMA and the HAMD, both of which have been extensively validated in Chinese clinical populations. Sleep quality was assessed using the PSQI, a widely used self‐report measure with well‐documented psychometric properties, including good internal consistency and test–retest reliability in Chinese populations [41]. Eye‐tracking and grasping measures were expressed as relative indices to account for individual differences in overall viewing time and motor activity. For eye‐tracking data, the fixation time ratio was calculated as the total fixation duration on alcohol‐related regions of interest divided by the total fixation duration across all regions of interest during the task, and the fixation count ratio was calculated as the number of fixations on alcohol‐related regions of interest divided by the total number of fixations across all regions of interest. For grasping behaviour, the grasping time ratio was calculated as the cumulative duration of grasping alcohol‐related virtual objects divided by the total task duration, and the grasping count ratio was calculated as the number of grasping events directed toward alcohol‐related virtual objects divided by the total number of grasping events during the task. These ratio‐based measures were used to provide normalized indices of attentional bias and approach behaviour toward alcohol‐related cues.

#### 2.3.2 Abstinence Status and Alcohol Use Control

All participants were hospitalized patients with AD and were abstinent at the beginning of the intervention following routine inpatient withdrawal management. During the intervention period, participants resided in a closed inpatient ward where alcohol consumption was strictly prohibited. Therefore, no alcohol use occurred during the intervention period, and no self‐report or biochemical verification of alcohol consumption was required.

#### 2.3.3 Procedure

VR‐CET procedure: VR‐CET was administered by a psychiatrist trained in the use of VR technology. The researcher briefly introduced the procedure to the patient and provided simple training on how to wear and use the VR device. The head strap and interpupillary distance of the VR headset were adjusted according to the patient's head circumference and eye spacing to ensure comfort and clear vision. Patients were instructed to stand, extend their arms and slowly move toward the dining table. They could freely observe or pick up alcohol‐related or non‐alcohol‐related cues according to their preference. During the session, the researcher sprayed a small amount of liquor into the air to provide olfactory stimulation. Throughout the treatment, the researcher closely monitored the patient to prevent collisions or falls. At the end of the session, the researcher informed the patient that the treatment had concluded, removed the VR headset, disinfected the device with an alcohol wipe and advised the patient to rest seated for a period before standing up to avoid dizziness caused by visual discomfort.

VR‐AT procedure: VR‐AT was also conducted by a psychiatrist trained in VR use. The researcher introduced the procedure and trained the patient in the use of the VR equipment. The VR headset was adjusted for comfort and clarity. Patients were assisted to sit comfortably in a chair, asked to relax and informed that the upcoming video content might cause discomfort. During viewing, patients were instructed to continuously adjust their perspective to align with the protagonist's viewpoint in the video. The researcher carefully monitored the patient throughout the session to prevent accidents. After completion, the researcher informed the patient that the treatment was finished, removed and disinfected the VR headset and advised the patient to rest before leaving to avoid dizziness.

Group interventions: After completion of acute withdrawal treatment, participants proceeded according to their assigned study group (control, VR‐CET, VR‐AT or VR‐CET + AT).

Control group: Received standard clinical treatment and viewed the VR relaxation scenario. Standard treatment primarily consisted of benzodiazepine substitution therapy during the early phase of hospitalization, supplemented with adequate B vitamins and, depending on clinical status, antipsychotics, antidepressants or mood stabilizers, together with symptomatic supportive care. Benzodiazepines were tapered after the disappearance of withdrawal symptoms. Patients viewed the VR relaxation scenario for 15 days, eight sessions in total, once every other day, approximately 10 min per session. This condition served as a neutral comparator by controlling for non‐specific effects of VR exposure while excluding the active therapeutic components of cue exposure and aversive learning. In addition, one VR‐CET session was added at both the first and last sessions to collect eye‐tracking and grasping data.

VR‐CET group: Received standard clinical treatment plus VR‐CET. Treatment lasted 15 days with eight sessions in total, once every other day, approximately 10 min per session. The duration of each cue‐exposure session was set at 10 min. This duration was selected to balance effective induction of cue‐related responses with participant safety and tolerability in an immersive VR environment. Previous VR‐based cue exposure studies have shown that relatively brief exposure periods are sufficient to elicit craving and attentional responses, whereas longer sessions may increase the risk of discomfort, fatigue or cybersickness, particularly in clinical populations [13]. Therefore, a 10‐min exposure was considered an appropriate and feasible duration for repeated inpatient interventions. Patients could freely move around in the restaurant‐style exposure scenario and grasp alcohol‐related or non‐alcohol‐related cues. During treatment, the alcohol craving assessment system we developed (Patent No. ZL 2024 11985775.9) recorded both eye‐tracking and grasping data.

VR‐AT group: Received standard clinical treatment plus VR‐AT. Treatment lasted 15 days with eight sessions in total, once every other day, approximately 10 min per session. The session duration was primarily determined by the length of the aversive video presented to the patient during the intervention. One VR‐CET session was also added at both the first and last sessions for eye‐tracking and grasping data collection. Four different aversive scenario videos were played, each shown twice.

**FIGURE 1:**
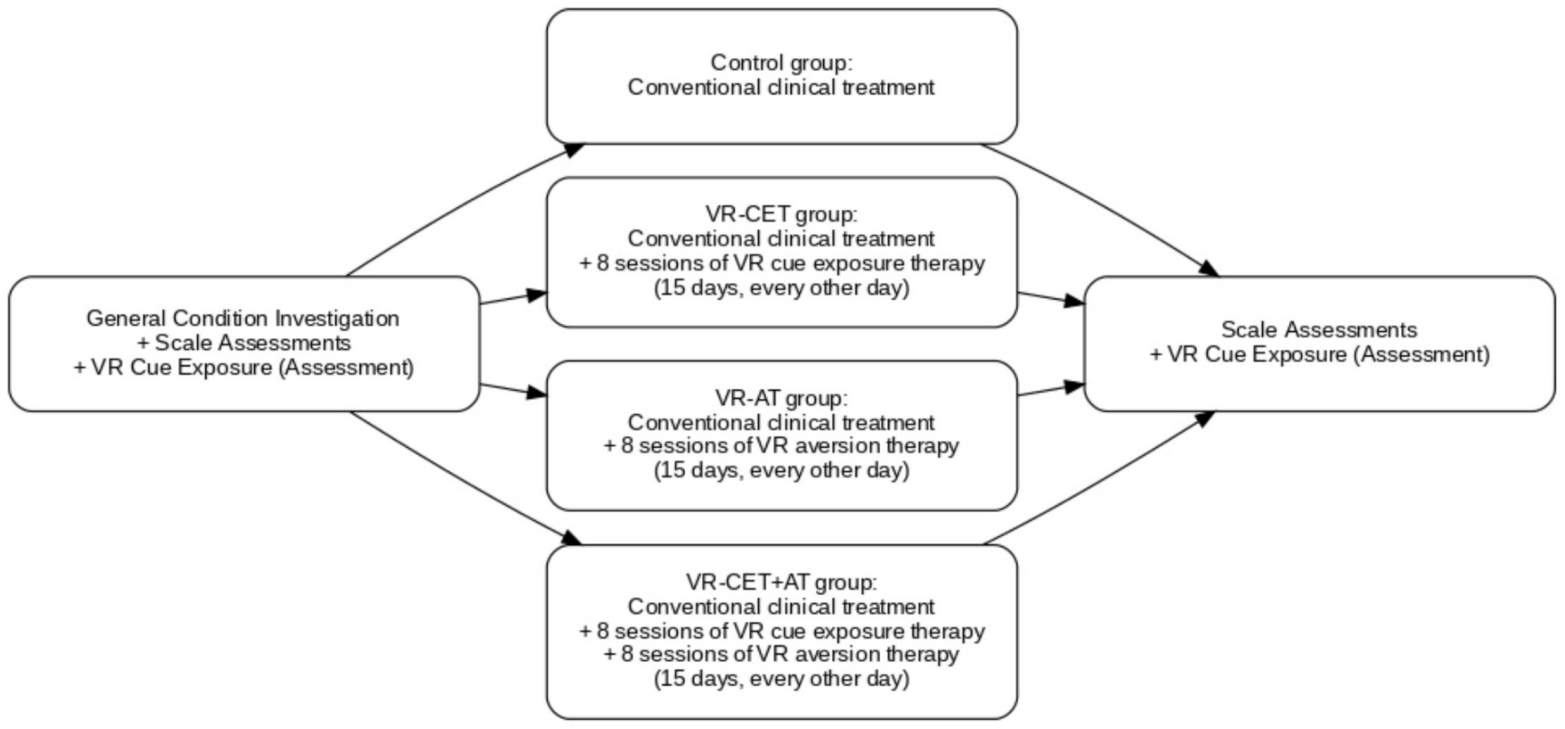
Flow chart of test procedure.

VR‐CET + AT group: Received standard clinical treatment combined with VR‐CET and VR‐AT. Treatment lasted 15 days with eight sessions in total, once every other day, approximately 20 min per session. Each session included one VR‐CET and one VR‐AT treatment, as shown in Figure 1.

During all VR sessions, participants were monitored for VR‐related discomfort or adverse events, including dizziness, nausea, visual discomfort and accidental injury.

#### 2.3.4 Assessment Time Points

Baseline assessments were completed at enrollment and included basic demographic and clinical information. CIWA‐Ar, VAS, PACS, HAMA, HAMD and PSQI were assessed at baseline and again 1 day after completion of the intervention. Relapse status was assessed only at 4 and 12 weeks after the intervention by standardized telephone follow‐up.

Eye‐tracking and grasping data were collected during the first and final treatment sessions. All questionnaire assessments and telephone follow‐up interviews were conducted by a single trained clinical psychiatrist who was not involved in group allocation or intervention delivery, thereby ensuring that outcome assessments were performed under blinded conditions.

#### 2.3.5 Outcomes

The primary endpoint of this randomized controlled trial was the change in alcohol craving following the intervention, as assessed by craving‐related measures. This endpoint was selected a priori because reduction in craving is a core therapeutic target in AD and was the basis for the sample size estimation. Secondary outcomes included changes in anxiety, depression, sleep quality, attentional bias, approach behaviour toward alcohol cues and relapse status assessed by standardized telephone follow‐up at 4 and 12 weeks.

#### 2.3.6 Statistical Method

Data were analysed using SPSS Version 29.0. Categorical variables were compared using the chi‐square test when the expected cell count in each cell was ≥ 5; otherwise, Fisher's exact test was used. For continuous variables, normality was assessed using the Shapiro–Wilk test. Normally distributed data are presented as mean ± standard deviation (x ± s), whereas non‐normally distributed data are presented as median (interquartile range) (M [P25, P75]).

For between‐group comparisons, one‐way ANOVA was used for normally distributed variables, followed by Tukey's honestly significant difference (HSD) test for post hoc pairwise comparisons when appropriate. Because Tukey's HSD inherently controls the family‐wise error rate, no additional multiplicity correction was applied for these comparisons. For non‐normally distributed variables, the Kruskal–Wallis test was used, followed by Dunn's post hoc test with Bonferroni‐adjusted *p* values for pairwise comparisons when appropriate. Thus, all reported post hoc pairwise comparisons were based on multiplicity‐adjusted *p* values.

Given the exploratory nature of this randomized controlled trial and the limited sample size in each group, the primary analyses focused on within‐group changes over time and between‐group differences at specific assessment time points. Accordingly, paired‐samples *t*‐tests or Wilcoxon signed‐rank tests were used to assess within‐group changes, depending on data distribution, and one‐way ANOVA or non‐parametric equivalents were used to compare outcomes between groups at each time point. Effect sizes were calculated and reported where appropriate to aid interpretation of the magnitude of observed effects. A two‐sided *p* value < 0.05 was considered statistically significant.

Although more complex approaches such as repeated‐measures ANOVA, mixed‐effects models or generalized estimating equations may better account for within‐ and between‐subject effects simultaneously, these methods were not adopted in the present analysis because of the limited sample size and the focus on clinically interpretable comparisons at specific time points.

## 3 Results

### 3.1 General Information

A total of 80 patients completed the study, with 20 patients in the control group, 20 in the VR‐CET group, 20 in the VR‐AT group and 20 in the VR‐CET + AT group. Five patients in the control group, six in the VR‐CET group, five in the VR‐AT group and five in the VR‐CET + AT group were excluded. All dropouts occurred because the patients were discharged during the course of the study. No VR‐related adverse events or clinically significant discomfort were observed during the intervention period, and no participant discontinued the study because of VR‐related adverse effects.

Baseline demographic and clinical characteristics of participants in the four groups are summarized in Table 1. Daily alcohol intake was calculated based on self‐reported average daily alcohol consumption prior to hospital admission. There were no statistically significant differences among the control, VR‐AT, VR‐CET and VR‐CET + AT groups with respect to age, height, weight, smoking‐related variables, duration of alcohol use or daily alcohol intake at baseline (all *p* > 0.05).

**TABLE 1:** Baseline demographic and clinical characteristics of participants.

	Control group (*n* = 20)	VR‐AT group (*n* = 20)	VR‐CET group (*n* = 20)	VR‐CET + AT group (*n* = 20)	*χ* ^2^/*F*	*p*
Age (years)	36.4 ± 7.2	39.0 ± 4.1	38.3 ± 9.1	40.2 ± 5.3	1.13	0.341
Height (cm)	173.0 ± 3.2	172.3 ± 4.0	171.2 ± 3.7	173.4 ± 5.2	1.16	0.329
Weight (kg)	66.3 ± 6.2	72.2 ± 11.7	67.2 ± 9.0	70.5 ± 7.1	1.98	0.124
Smoking duration (years)	15.0 (10.0, 20.0)	15.0 (10.0, 18.5)	15.0 (13.8, 20.0)	10.0 (10.0, 15.0)	6.62	0.085
Cigarettes per day	20.0 (10.0, 20.0)	20.0 (15.0, 20.0)	16.5 (10.0, 20.0)	14.5 (10.0, 20.0)	3.85	0.279
Total drinking duration (years)	16.0 (12.8, 20.0)	17.5 (15.0, 19.0)	15.0 (13.8, 16.2)	13.0 (10.0, 15.2)	7.19	0.066
Daily alcohol intake (g)	209 (162 251)	209 (183 209)	139 (115 209)	157 (120 251)	4.26	0.235

*Note:* Data are presented as mean ± standard deviation (SD) for normally distributed variables and as median (interquartile range) for non‐normally distributed variables. *χ* ^2^/*F* represents the test statistic from the chi‐square test or one‐way analysis of variance, as appropriate.

Abbreviations: VR‐AT, virtual reality–based aversion therapy; VR‐CET, virtual reality–based cue exposure therapy; VR‐CET + AT, combined virtual reality–based cue exposure and aversion therapy.

### 3.2 Comparison of Pre‐Treatment Scale Assessments, Eye‐Tracking Data and Grasping Data Among the Four Groups

Before treatment, no statistically significant differences were found among the four groups in the ratios of alcohol cue fixation time, alcohol cue fixation counts, alcohol cue grasping time, alcohol cue grasping counts, PACS scores, HAMD scores, HAMA scores, PSQI scores or VAS scores (all *p* > 0.05), as shown in Table 2.

**TABLE 2:** Comparison of baseline data across the four groups.

	Control (*n* = 20)	VR‐AT (*n* = 20)	VR‐CET (*n* = 20)	VR‐CET + AT (*n* = 20)	*F*/*H*	*p*
Alcohol cue fixation time ratio	0.66 ± 0.14	0.70 ± 0.06	0.65 ± 0.11	0.65 ± 0.09	0.899	0.446
Alcohol cue fixation count ratio	0.67 ± 0.11	0.72 ± 0.04	0.66 ± 0.06	0.67 ± 0.07	2.612	0.057
Alcohol cue grasping time ratio	0.53 ± 0.22	0.45 ± 0.19	0.51 ± 0.21	0.53 ± 0.27	0.528	0.664
Alcohol cue grasping count ratio	0.54 ± 0.20	0.49 ± 0.18	0.57 ± 0.25	0.49 ± 0.27	0.548	0.651
PACS score	20.75 ± 6.50	20.45 ± 4.49	18.80 ± 4.21	20.95 ± 2.95	0.865	0.463
HAMD score	14.80 ± 6.09	14.75 ± 6.54	15.30 ± 5.77	14.70 ± 5.42	0.043	0.988
HAMA score	11.90 ± 5.02	12.75 ± 4.29	13.60 ± 5.48	12.75 ± 5.79	0.360	0.782
PSQI score	8.00 (7.00, 10.00)	7.00 (5.00, 10.25)	6.00 (5.00, 9.25)	9.00 (6.75, 10.50)	3.552	0.314
VAS score	7.50 (7.00, 8.00)	7.00 (6.00, 7.25)	7.00 (7.00, 8.00)	7.00 (6.75, 8.00)	5.076	0.166

*Note:* Data are presented as mean ± standard deviation (SD) for normally distributed variables and as median (interquartile range) for non‐normally distributed variables. *F*/*H* represents the test statistic from one‐way analysis of variance or the Kruskal–Wallis test, as appropriate.

Abbreviations: HAMA, Hamilton Anxiety Scale; HAMD, Hamilton Depression Scale; PACS, Pennsylvania Alcohol Craving Scale; PSQI, Pittsburgh Sleep Quality Index; VAS, Visual Analogue Scale; VR‐AT, virtual reality–based aversion therapy; VR‐CET, virtual reality–based cue exposure therapy; VR‐CET + AT, combined virtual reality–based cue exposure and aversion therapy.

### 3.3 Comparison of Pre‐ and Post‐Treatment Scale Assessments, Eye‐Tracking Data and Grasping Data Across the Four Groups

The PACS, HAMD, HAMA, PSQI and VAS scores showed significant differences before and after treatment across the four groups (*p* < 0.001), whereas no statistically significant differences were observed in the other measures before and after treatment (*p* > 0.05), as shown in Tables 3, 4, 5, 6.

**TABLE 3:** The comparison of pre‐ and post‐treatment data in the control group.

	Before treatment (*n* = 20)	After treatment (*n* = 20)	*t*/*Z*	*p*
Alcohol cue fixation time ratio	0.66 ± 0.14	0.71 ± 0.15	−1.435	0.168
Alcohol cue fixation count ratio	0.67 ± 0.11	0.69 ± 0.12	−0.783	0.443
Alcohol cue grasping time ratio	0.53 ± 0.22	0.52 ± 0.20	0.248	0.807
Alcohol cue grasping count ratio	0.54 ± 0.20	0.53 ± 0.17	0.027	0.979
PACS score	20.75 ± 6.50	8.25 ± 4.36	8.127	< 0.001
HAMD score	14.80 ± 6.09	4.50 ± 1.67	7.545	< 0.001
HAMA score	11.90 ± 5.02	3.50 ± 2.40	6.479	< 0.001
PSQI score	8.00 (7.00, 10.00)	3.00 (2.00, 5.00)	−3.461	< 0.001
VAS score	7.50 (7.00, 8.00)	3.50 (1.00, 6.00)	−3.179	< 0.001

*Note:* Data are presented as mean ± standard deviation (SD) for normally distributed variables and as median (interquartile range) for non‐normally distributed variables. *t*/*Z* represents the test statistic from the paired‐samples *t*‐test or the Wilcoxon signed‐rank test, as appropriate.

Abbreviations: HAMA, Hamilton Anxiety Scale; HAMD, Hamilton Depression Scale; PACS, Pennsylvania Alcohol Craving Scale; PSQI, Pittsburgh Sleep Quality Index; VAS, Visual Analogue Scale.

**TABLE 4:** The comparison of pre‐ and post‐treatment data in the VR‐AT group.

	Before treatment (*n* = 20)	After treatment (*n* = 20)	*t*/*Z*	*p*
Alcohol cue fixation time ratio	0.70 ± 0.06	0.67 ± 0.14	1.135	0.271
Alcohol cue fixation count ratio	0.72 ± 0.04	0.69 ± 0.09	1.413	0.174
Alcohol cue grasping time ratio	0.45 ± 0.19	0.48 ± 0.27	−0.593	0.560
Alcohol cue grasping count ratio	0.49 ± 0.18	0.45 ± 0.23	0.691	0.498
PACS score	20.45 ± 4.49	6.60 ± 2.04	12.922	< 0.001
HAMD score	14.75 ± 6.54	4.50 ± 2.74	9.753	< 0.001
HAMA score	12.75 ± 4.29	3.45 ± 1.61	11.649	< 0.001
PSQI score	7.00 (5.00, 10.25)	4.00 (1.00, 6.00)	−3.361	< 0.001
VAS score	7.00 (6.00, 7.25)	1.00 (0.00, 3.00)	−3.920	< 0.001

*Note:* Data are presented as mean ± standard deviation (SD) for normally distributed variables and as median (interquartile range) for non‐normally distributed variables. *t*/*Z* represents the test statistic from the paired‐samples *t*‐test or the Wilcoxon signed‐rank test, as appropriate.

Abbreviations: HAMA, Hamilton Anxiety Scale; HAMD, Hamilton Depression Scale; PACS, Pennsylvania Alcohol Craving Scale; PSQI, Pittsburgh Sleep Quality Index; VAS, Visual Analogue Scale.

**TABLE 5:** The comparison of pre‐ and post‐treatment data in the VR‐CET group.

	Before treatment (*n* = 20)	After treatment (*n* = 20)	*t*/*Z*	*p*
Alcohol cue fixation time ratio	0.65 ± 0.11	0.59 ± 0.15	1.574	0.132
Alcohol cue fixation count ratio	0.66 ± 0.06	0.62 ± 0.11	1.296	0.211
Alcohol cue grasping time ratio	0.51 ± 0.21	0.47 ± 0.27	0.493	0.628
Alcohol cue grasping count ratio	0.57 ± 0.25	0.55 ± 0.22	0.236	0.816
PACS score	18.80 ± 4.21	7.50 ± 3.78	12.31	< 0.001
HAMD score	15.30 ± 5.77	4.00 ± 2.18	9.733	< 0.001
HAMA score	13.60 ± 5.48	2.95 ± 1.40	9.152	< 0.001
PSQI score	6.00 (5.00, 9.25)	2.00 (2.00, 5.25)	−3.584	< 0.001
VAS score	7.00 (7.00, 8.00)	2.00 (2.00, 3.00)	−3.920	< 0.001

*Note:* Data are presented as mean ± standard deviation (SD) for normally distributed variables and as median (interquartile range) for non‐normally distributed variables. *t*/*Z* represents the test statistic from the paired‐samples *t*‐test or the Wilcoxon signed‐rank test, as appropriate.

Abbreviations: HAMA, Hamilton Anxiety Scale; HAMD, Hamilton Depression Scale; PACS, Pennsylvania Alcohol Craving Scale; PSQI, Pittsburgh Sleep Quality Index; VAS, Visual Analogue Scale.

**TABLE 6:** The comparison of pre‐ and post‐treatment data in the VR‐CET + AT group.

	Before treatment (*n* = 20)	After treatment (*n* = 20)	*t*/*Z*	*p*
Alcohol cue fixation time ratio	0.65 ± 0.09	0.57 ± 0.18	2.424	0.026*
Alcohol cue fixation count ratio	0.67 ± 0.07	0.63 ± 0.13	1.196	0.246
Alcohol cue grasping time ratio	0.53 ± 0.27	0.50 ± 0.23	0.420	0.678
Alcohol cue grasping count ratio	0.49 ± 0.27	0.50 ± 0.26	−0.167	0.869
PACS score	20.95 ± 2.95	6.55 ± 2.50	16.405	< 0.001
HAMD score	14.70 ± 5.42	3.55 ± 1.57	9.349	< 0.001
HAMA score	12.75 ± 5.79	2.75 ± 1.33	7.185	< 0.001
PSQI score	9.00 (6.75, 10.50)	2.00 (0.00, 6.00)	−3.808	< 0.001
VAS score	7.00 (6.75, 8.00)	1.00 (0.75, 2.00)	−3.920	< 0.001

*Note:* Data are presented as mean ± standard deviation (SD) for normally distributed variables and as median (interquartile range) for non‐normally distributed variables. *t*/*Z* represents the test statistic from the paired‐samples *t*‐test or the Wilcoxon signed‐rank test, as appropriate.

Abbreviations: HAMA, Hamilton Anxiety Scale; HAMD, Hamilton Depression Scale; PACS, Pennsylvania Alcohol Craving Scale; PSQI, Pittsburgh Sleep Quality Index; VAS, Visual Analogue Scale.

* *p* < 0.05.

### 3.4 Comparison of Post‐Treatment Scale Assessments, Eye‐Tracking Data and Grasping Data Among the Four Groups

After treatment, the alcohol cue fixation time ratio showed a statistically significant overall between‐group difference (*p* < 0.05). Tukey's HSD‐adjusted post hoc comparisons revealed a significant difference between the control group and the VR‐CET + AT group (adjusted *p* = 0.019). The VAS score also showed a statistically significant overall between‐group difference (*p* < 0.05); Dunn's post hoc comparisons with Bonferroni‐adjusted *p* values indicated a significant difference between the control group and the VR‐CET + AT group (adjusted *p* = 0.008). No other pairwise comparisons reached statistical significance, as shown in Tables 7, 8, 9.

**TABLE 7:** The comparison of post‐treatment data among the four groups.

	Control (*n* = 20)	VR‐AT (*n* = 20)	VR‐CET (*n* = 20)	VR‐CET+AT (*n* = 20)	*F*/*H*	*p*
Alcohol cue fixation time ratio	0.71 ± 0.15	0.67 ± 0.14	0.59 ± 0.15	0.57 ± 0.18	3.934	0.012*
Alcohol cue fixation count ratio	0.69 ± 0.12	0.69 ± 0.09	0.62 ± 0.11	0.63 ± 0.13	2.067	0.112
Alcohol cue grasping time ratio	0.52 ± 0.20	0.48 ± 0.27	0.47 ± 0.27	0.34 ± 0.20	2.206	0.094
Alcohol cue grasping count ratio	0.53 ± 0.17	0.45 ± 0.23	0.51 ± 0.24	0.51 ± 0.29	0.400	0.753
PACS score	8.25 ± 4.36	6.60 ± 2.04	7.50 ± 3.78	6.55 ± 2.50	1.203	0.314
HAMD score	4.50 ± 1.67	4.50 ± 2.74	4.00 ± 2.18	3.55 ± 1.57	0.954	0.419
HAMA score	3.50 ± 2.40	3.45 ± 1.61	2.95 ± 1.40	2.75 ± 1.33	0.913	0.439
PSQI score	3.00 (2.00, 5.00)	4.00 (1.00, 6.00)	2.00 (2.00, 5.30)	2.00 (0.00, 6.00)	4.197	0.241
VAS score	3.50 (1.00, 6.00)	1.00 (0.00, 3.00)	2.00 (2.00, 3.00)	1.00 (0.80, 2.00)	13.144	0.004**

*Note:* Data are presented as mean ± standard deviation (SD) for normally distributed variables and as median (interquartile range) for non‐normally distributed variables. *F*/*H* represents the test statistic from one‐way analysis of variance or the Kruskal–Wallis test, as appropriate.

Abbreviations: HAMA, Hamilton Anxiety Scale; HAMD, Hamilton Depression Scale; PACS, Pennsylvania Alcohol Craving Scale; PSQI, Pittsburgh Sleep Quality Index; VAS, Visual Analogue Scale; VR‐AT, virtual reality–based aversion therapy; VR‐CET, virtual reality–based cue exposure therapy; VR‐CET + AT, combined virtual reality–based cue exposure and aversion therapy.

* *p* < 0.05.

** *p* < 0.01.

**TABLE 8:** Post hoc test of alcohol cue fixation time ratio.

Comparison	Mean difference	Adjusted *p*
VR‐AT group vs. VR‐CET + AT group	−0.099	0.184
VR‐AT group vs. VR‐CET group	−0.080	0.361
VR‐AT group vs. control group	0.046	0.775
VR‐CET + AT group vs. VR‐CET group	0.019	0.980
VR‐CET + AT group vs. control group	0.145	0.019*
VR‐CET group vs. control group	0.126	0.054

*Note:* Post hoc comparisons were performed using Tukey's honestly significant difference (HSD) test following one‐way analysis of variance. Tukey's HSD controls the family‐wise error rate, and the reported *p* values are multiplicity‐adjusted.

Abbreviations: VR‐AT, virtual reality–based aversion therapy; VR‐CET, virtual reality–based cue exposure therapy; VR‐CET + AT, combined virtual reality–based cue exposure and aversion therapy.

* *p* < 0.05.

**TABLE 9:** Post hoc test of VAS score.

Comparison	Mean rank difference	Adjusted *p*
Control group vs. VR‐AT group	15.925	0.181
Control group vs. VR‐CET group	4.975	1.000
Control group vs. VR‐CET + AT group	23.600	0.008**
VR‐AT group vs. VR‐CET group	10.950	0.817
VR‐AT group vs. VR‐CET + AT group	7.675	1.000
VR‐CET + AT group vs. VR‐CET group	18.625	0.068

*Note:* Post hoc comparisons were performed using Dunn's test following the Kruskal–Wallis test. Reported *p* values are adjusted for multiple comparisons using the Bonferroni method.

Abbreviations: VAS, Visual Analogue Scale; VR‐AT, virtual reality–based aversion therapy; VR‐CET, virtual reality–based cue exposure therapy; VR‐CET + AT, combined virtual reality–based cue exposure and aversion therapy.

** *p* < 0.01.

### 3.5 Comparison of Pre‐ and Post‐Treatment Differences in Scale Assessments, Eye‐Tracking Data and Grasping Data Among the Four Groups

The between‐group difference in the change of alcohol cue fixation time ratio was statistically significant (*p* < 0.05). Tukey's HSD‐adjusted post hoc comparisons showed that the difference between the control group and the VR‐CET + AT group remained statistically significant (adjusted *p* = 0.040). The between‐group difference in the change of VAS score was also statistically significant at the omnibus level (*p* < 0.05); however, Dunn's post hoc comparisons with Bonferroni‐adjusted *p* values showed that none of the pairwise comparisons reached statistical significance, as shown in Tables 10, 11, 12.

**TABLE 10:** The comparison of pre‐ and post‐treatment differences among the groups.

	Control group (*n* = 20)	VR‐AT group (*n* = 20)	VR‐CET group (*n* = 20)	VR‐CET + AT group (*n* = 20)	*F*/*H*	*p*
Difference in alcohol cue fixation time ratio	−0.05 ± 0.15	0.03 ± 0.13	0.06 ± 0.18	0.09 ± 0.16	2.844	0.043*
Difference in alcohol cue fixation count ratio	−0.02 ± 0.13	0.04 ± 0.09	0.04 ± 0.13	0.04 ± 0.14	1.139	0.339
Difference in alcohol cue grasping time ratio	0.02 ± 0.31	−0.03 ± 0.23	0.03 ± 0.31	0.19 ± 0.27	2.324	0.082
Difference in alcohol cue grasping count ratio	0.00 ± 0.25	0.04 ± 0.24	0.06 ± 0.29	−0.02 ± 0.36	0.293	0.830
Difference in PACS score	12.50 ± 6.88	13.85 ± 4.79	11.30 ± 4.11	14.40 ± 3.93	1.514	0.218
Difference in HAMD score	10.30 ± 6.11	10.25 ± 4.70	11.30 ± 5.19	11.15 ± 5.33	0.213	0.887
Difference in HAMA score	8.40 ± 5.80	9.30 ± 3.57	10.65 ± 5.20	10.00 ± 6.22	0.664	0.577
Difference in PSQI score	4.00 (1.80, 6.00)	4.50 (0.80, 5.30)	3.00 (2.00, 5.00)	6.50 (2.50, 9.30)	4.288	0.232
Difference in VAS score	4.50 (1.00, 6.30)	5.00 (3.80, 6.00)	5.00 (4.00, 6.00)	6.00 (6.00, 6.30)	8.867	0.031*

*Note:* Data are presented as mean ± standard deviation (SD) for normally distributed variables and as median (interquartile range) for non‐normally distributed variables. Differences represent post‐treatment values minus baseline values. *F*/*H* represents the test statistic from one‐way analysis of variance or the Kruskal–Wallis test, as appropriate.

Abbreviations: HAMA, Hamilton Anxiety Scale; HAMD, Hamilton Depression Scale; PACS, Pennsylvania Alcohol Craving Scale; PSQI, Pittsburgh Sleep Quality Index; VAS, Visual Analogue Scale; VR‐AT, virtual reality–based aversion therapy; VR‐CET, virtual reality–based cue exposure therapy; VR‐CET + AT, combined virtual reality–based cue exposure and aversion therapy.

* *p* < 0.05.

**TABLE 11:** Post hoc test of alcohol cue fixation time ratio difference.

Comparison	Mean difference	Adjusted *p*
VR‐AT group vs. VR‐CET + AT group	0.054	0.586
VR‐AT group vs. VR‐CET group	−0.001	1.000
VR‐AT group vs. control group	−0.079	0.249
VR‐CET + AT group vs. VR‐CET group	−0.054	0.576
VR‐CET + AT group vs. control group	−0.133	0.040*
VR‐CET group vs. control group	−0.079	0.255

*Note:* Post hoc comparisons were performed using Tukey's honestly significant difference (HSD) test following one‐way analysis of variance. Tukey's HSD controls the family‐wise error rate, and the reported *p* values are multiplicity‐adjusted.

Abbreviations: VR‐AT, virtual reality–based aversion therapy; VR‐CET, virtual reality–based cue exposure therapy; VR‐CET + AT, combined virtual reality–based cue exposure and aversion therapy.

* *p* < 0.05.

**TABLE 12:** Post hoc test of VAS score difference.

Comparison	Mean rank difference	Adjusted *p*
Control group vs. VR‐AT group	0.625	1.000
Control group vs. VR‐CET group	5.250	1.000
Control group vs. VR‐CET + AT group	18.825	0.062
VR‐AT group vs. VR‐CET group	4.625	1.000
VR‐AT group vs. VR‐CET + AT group	18.200	0.080
VR‐CET group vs. VR‐CET + AT group	13.575	0.388

*Note:* Post hoc comparisons were performed using Dunn's test following the Kruskal–Wallis test. Reported *p* values are adjusted for multiple comparisons using the Bonferroni method.

Abbreviations: VAS, Visual Analogue Scale; VR‐AT, virtual reality–based aversion therapy; VR‐CET, virtual reality–based cue exposure therapy; VR‐CET + AT, combined virtual reality–based cue exposure and aversion therapy.

### 3.6 The Comparison of Relapse Among the Four Groups After Treatment

Telephone follow‐up at 4 and 12 weeks post‐treatment was completed in 47 patients. As some expected counts were < 5, Fisher's exact test was applied for between‐group comparisons. No significant differences were observed (*p* > 0.05), as shown in Table 13.

**TABLE 13:** Comparison of relapse among the four groups at 4 and 12 weeks.

	Control group (*n* = 20)	VR‐AT group (*n* = 20)	VR‐CET group (*n* = 20)	VR‐CET + AT group (*n* = 20)	*p*
Relapse at 4 weeks					0.68
Relapse, *n*	8	6	4	4	
Abstinence, *n*	5	5	8	7	
Lost to follow‐up, *n*	7	9	8	9	
Relapse rate, % (ITT)	75.0	75.0	60.0	65.0	
Relapse at 12 weeks					0.89
Relapse, *n*	10	9	8	8	
Abstinence, *n*	3	2	4	3	
Lost to follow‐up, *n*	7	9	8	9	
Relapse rate, % (ITT)	85.0	90.0	80.0	85.0	

*Note:* Relapse rates were calculated according to the intention‐to‐treat (ITT) principle, with participants lost to follow‐up conservatively classified as relapsed. *p* values were calculated using Fisher's exact test.

Abbreviations: VR‐AT, virtual reality–based aversion therapy; VR‐CET, virtual reality–based cue exposure therapy; VR‐CET + AT, combined virtual reality–based cue exposure and aversion therapy.

## 4 Discussion

### 4.1 Main Findings

In this randomized controlled trial, we compared the effects of VR‐CET, VR‐AT, their combination (VR‐CET + AT) and a control condition involving relaxation training among patients with ad. All groups, including controls, showed significant improvements in PACS, HAMD, HAMA, VAS and PSQI scores after intervention, suggesting that inpatient care and routine treatment alone may yield therapeutic benefits [42, 43]. However, greater improvements were observed in the VR intervention groups, particularly in the VR‐CET + AT group, which demonstrated more pronounced reductions in VAS scores and alcohol cue fixation time compared with controls. These results indicate potential added benefits of combining cue exposure with AT [13]. With respect to relapse outcomes, intention‐to‐treat analyses revealed no statistically significant differences among the four groups at either Week 4 or Week 12. Although descriptive differences in relapse rates were observed at the early follow‐up, these findings should be interpreted with caution given the limited sample size, substantial loss to follow‐up and the relatively short follow‐up duration [44]. Therefore, the present study mainly supports the effects of VR‐based interventions at the level of craving reduction and cue‐related attentional bias, whereas larger studies with longer follow‐up are needed to determine whether these mechanism‐level improvements translate into robust relapse prevention benefits.

### 4.2 Possible Mechanisms

#### 4.2.1 CET

Craving in ad patients is often the result of conditioned learning, in which repeated alcohol use creates strong associations between alcohol‐related cues (e.g., bottles, restaurants) and rewarding effects. VR‐CET is grounded in extinction learning, whereby repeated exposure to alcohol‐related stimuli without actual drinking gradually weakens the cue–craving association [45, 46, 47]. In this study, both the VR‐CET and VR‐CET + AT groups showed significant reductions in PACS and VAS scores post‐intervention, and the VR‐CET + AT group demonstrated significant improvements compared with controls, supporting the effectiveness of extinction‐based mechanisms. Unlike traditional exposure, VR provides a safe and highly immersive environment in which patients can rehearse high‐risk scenarios, thereby facilitating habituation and attenuation of craving responses [48].

#### 4.2.2 AT

The principle of AT lies in establishing a conditioned association between alcohol use and negative consequences, thereby reducing drinking desire [49]. Traditional methods such as electrical shocks or emetic drugs have demonstrated efficacy but are limited by poor safety and adherence [50]. In this study, we developed multiple VR‐based aversive scenarios (e.g., quarrelling with a partner, hypothermia after drinking, cycling accidents, vomiting), allowing patients to experience discomfort and fear in an immersive yet safe environment. These ‘virtual aversive stimuli’ avoided severe physiological harm while still eliciting strong negative emotional responses, thereby contributing to the suppression of craving [51]. Previous studies have shown that AT may alter reward‐related neural circuits and promote avoidance responses toward alcohol cues [52]. Consistent with this, both the VR‐AT and VR‐CET + AT groups exhibited significant reductions in PACS and VAS scores. Although the VR‐AT group did not differ significantly from controls in post‐intervention VAS, the VR‐CET + AT group showed significant improvements, suggesting that VR‐AT may play a contributing role in reducing craving. The lack of significance in the VR‐AT group alone may be due to limited sample size.

#### 4.2.3 Synergistic Effects of Combined Intervention

The superior outcomes in the VR‐CET + AT group suggest potential synergistic effects of combining cue exposure and AT. Cue exposure primarily weakens pre‐existing reward associations through extinction, whereas AT establishes new negative associations through conditioning. Together, these approaches may simultaneously reduce the attractiveness of alcohol cues and enhance negative expectancies, providing a dual protective mechanism [53, 54].

### 4.3 Comparison With Previous Studies

Previous studies have reported that VR‐CET can effectively reduce craving in patients with AUD. Zhang et al. demonstrated that VR‐CET significantly decreased VAS craving scores compared with conventional treatment [13]. Similarly, Hernández‐Serrano et al. found that outpatient AUD patients receiving TAU + VR‐CET showed significant reductions in craving, whereas those receiving TAU alone did not [55]. These findings align with our results, in which both VR‐CET and VR‐CET + AT significantly reduced PACS and VAS scores, with the VR‐CET + AT group showing significant differences compared with controls.

By contrast, relatively few recent studies have focused on AT, although earlier research confirmed its efficacy in reducing alcohol consumption and craving [56]. Elkins et al. reported that chemical AT reshaped reward circuits related to drinking behaviour [53]. Extending this line of research, our study combined VR with AT and demonstrated feasibility, safety and preliminary effectiveness in reducing craving and attentional bias, addressing limitations of traditional aversion methods.

With regard to relapse, a double‐blind RCT involving 300 AUD patients found that those receiving approach bias modification (ApBM) had significantly higher abstinence rates than controls at 3 months (OR ≈1.93, *p* = 0.012), although differences were not maintained at 6 and 12 months [57]. Similarly, although relapse rates at 4 weeks were lower in the VR groups in our study, differences were no longer evident by 12 weeks. These findings suggest that the short‐term effects of VR interventions may be more pronounced than long‐term outcomes, underscoring the need for larger samples and extended follow‐up to fully assess relapse prevention efficacy. At present, our results are more appropriately interpreted as supporting beneficial effects on proximal mechanisms of relapse risk, including craving and cue‐related attentional bias, rather than definitive evidence of relapse prevention.

### 4.4 Clinical Implications

This study provides preliminary evidence that VR‐based cue exposure and AT are safe, non‐invasive and well‐accepted interventions that may serve as adjunctive treatments for ad. Compared with traditional AT, VR‐AT avoids severe physical discomfort while still eliciting strong negative experiences, potentially improving treatment adherence. VR‐CET enables patients to rehearse coping skills in realistic high‐risk contexts, enhancing self‐control in real‐life situations. When combined, VR‐CET + AT appeared most effective in reducing craving and improving attentional measures, suggesting clinical value in relapse prevention. With increasing accessibility and affordability of VR technology, such interventions hold promise for wider clinical application.

### 4.5 Limitations and Future Directions

Several limitations should be acknowledged. First, the sample size was relatively small, and only male patients were included, limiting the generalizability of findings. Future studies should include larger and more diverse samples, including female patients. Second, the follow‐up period was limited to 12 weeks, precluding conclusions about long‐term relapse prevention. Extended follow‐up (≥ 6–12 months) with reduced attrition is needed. Relapse was assessed using a binary self‐reported measure, which does not capture drinking quantity, frequency or distinctions between lapse and relapse, and may therefore oversimplify the complexity of post‐treatment drinking behaviour. Quantitative measures of alcohol consumption (e.g., drinking amount or frequency) were not systematically collected as longitudinal outcomes, which limits the ability to evaluate changes in drinking behaviour beyond relapse status. In addition, the trial was registered retrospectively, which should be considered a limitation and may increase the risk of reporting bias. Third, objective measures were limited to eye‐tracking and grasping indices; incorporating additional physiological markers (e.g., HRV, skin conductance) and neuroimaging outcomes would provide more comprehensive insights. In addition, although repeated measurements were collected, more advanced longitudinal analytical approaches may further elucidate individual change trajectories and interaction effects in future studies with larger samples. Despite these limitations, this study provides an important foundation for developing VR‐based behavioural interventions for AD. Future research should focus on validating these findings across different populations, refining VR protocols and exploring the neural mechanisms underlying craving reduction.

## 5 Conclusion

In conclusion, the combination of VR‐CET and VR‐AT appeared to reduce craving intensity and attentional bias in patients with AD, whereas all treatment groups demonstrated clinical improvement in emotional and sleep outcomes. Although these findings suggest that integrating VR‐CET and VR‐AT may offer a promising adjunctive approach to conventional treatment, the evidence should be interpreted with caution given the limited sample size and short follow‐up duration. Future studies with larger and more diverse samples are needed to validate these results and to further clarify the mechanisms underlying the therapeutic effects of VR‐based interventions.

## Author Contributions

Haoyu Zhao, Wenhui Li, Hongdou Deng and Chuansheng Wang made substantial contributions to the conception and design of the work. Haoyu Zhao, Jingshu Zhang, Zihang Shao, Lanci Liu, Ming Chen, Genhui Ren, Jie Zhang, Ying Xu, Junjun Zhang and Ping Cui made substantial contributions to data acquisition. Haoyu Zhao, Dan Wang, Xinyou Wang, Xiaotong Ying and Xiaoyu Du contributed to data analysis and interpretation. Haoyu Zhao, Jiali Wang and Chunyan Li made substantial contributions to drafting the manuscript. Dan Wang contributed to the interpretation of results, provided critical revisions of important intellectual content and approved the final version of the manuscript. All authors have read and approved the final manuscript.

## Funding

This work was supported by the Open Project of Psychiatry and Neuroscience Discipline of Second Affiliated Hospital of Henan Medical University (XYEFYJSSJ‐2023‐03, XYEFYJSSJ‐2024‐06, XYEFYJSSJ‐2024‐01), the Henan Province science and technology research and development plan joint fund (industry) major project (235101610004), the Henan Provincial Science and Technology Research Project, China (242102310286, LHGJ20240507, LHGJ20240498), the 2024 Research and Innovation Support Program for Postgraduates, the Henan Medical University (YJSCX202432Y) and the Henan Provincial Key Laboratory of Addiction Medicine.

## Ethics Statement

The study protocol was reviewed and approved by the Ethics Committee of the Second Affiliated Hospital of Henan Medical University (XYEFYLL‐(Research)‐2024‐58) on 20/09/2024 and was conducted in accordance with the Declaration of Helsinki and subsequent revisions. That is, all experiments were conducted in accordance with relevant guidelines and regulations. All participants were fully informed of the purpose of the study and signed the informed consent form. Patients were informed that their participation was voluntary, and that they had the right to withdraw from the study at any time with no negative effect on their treatment. The study protocol was also registered at the China Clinical Trial Registry on 29/09/2025 (www.chictr.org.cn; ChiCTR ID: ChiCTR2500110026).

## Consent

The authors have nothing to report.

## Conflicts of Interest

The authors declare no conflicts of interest.

## Supporting information


**Figure S1:** Screenshots of the four aversive scenarios.
**Figure S2:** Filming equipment for aversion scenarios.
**Figure S3:** Screenshot of the cue‐exposure scenario.
**Figure S4:** Six relaxation scenarios.

## Data Availability

The datasets generated and analysed during the current study are not publicly available due to patient privacy regulations but are available from the corresponding authors upon reasonable request.
