# Supplementary material for: Virtual Reality–Based Cue Exposure and Aversion Therapy for Alcohol Dependence: A Randomized Controlled Trial
Source: Addict Biol. 2026 May 6;31(5):e70166. doi: 10.1111/adb.70166 (PMC13148881; doi:10.1111/adb.70166)
Supplement: Supplementary file 1 — Figure S1: Screenshots of the four aversive scenarios. Figure S2: Filming equipment for aversion scenarios. Figure S3: Screenshot of the cue‐exposure scenario. Figure S4: Six relaxation scenarios. [file ADB-31-e70166-s001.docx]

**Supporting Information**

**for**

**Virtual reality–based cue exposure and aversion therapy for alcohol dependence: a randomized controlled trial**

**Contents**

· Supplementary Methods S1. Development and validation of VR aversion scenarios

· Supplementary Methods S2. Development and validation of VR cue-exposure scenarios

· Supplementary Methods S3. Development and validation of VR relaxation scenarios

· Supplementary Questionnaire S1. Semi-structured interview questionnaire for aversion scenario development

· Figure S1. Screenshots of the four aversive scenarios

· Figure S2. Filming equipment for aversion scenarios

· Figure S3. Screenshot of the cue-exposure scenario

· Figure S4. Six relaxation scenarios

**Supplementary Methods S1. Development and validation of VR aversion scenarios**

**S1.1 Interview-based design rationale**

Because drinking cultures and lifestyles differ across countries, the severe consequences associated with long-term alcohol misuse may also vary. Therefore, in this study, virtual reality aversion scenarios were specifically designed to reflect the real-life contexts and characteristics of patients with alcohol dependence in China.

Between September 2023 and February 2024, researchers conducted semi-structured interviews with male patients with alcohol dependence who were admitted to Ward 5 of the Addiction Medicine Center at the Second Affiliated Hospital of Henan Medical University. These interviews were conducted during the formative phase of the study to inform scenario development and were independent of the subsequent randomization procedure. All participants met the diagnostic criteria for alcohol dependence according to the International Classification of Diseases, 10th Revision (ICD-10). The study protocol was retrospectively registered in the Chinese Clinical Trial Registry on September 29, 2025 (ChiCTR ID: ChiCTR2500110026) and was approved by the Ethics Committee of the Second Affiliated Hospital of Henan Medical University (Approval No. XYEFYLL-(Research)-2024-58).

The interviews collected basic demographic information (e.g., age, education, marital status, occupation, and drinking history) as well as clinical information relevant to scenario design, including typical drinking venues, most feared consequences of drinking, persons with whom participants were most unwilling to drink, and emotional states in situations when they most wished to avoid drinking. For these items, participants were allowed to choose a minimum of one and a maximum of three options. Responses were documented using a structured interview form. The full semi-structured interview questionnaire used for scenario development is provided as Supplementary Questionnaire 1.

**S1.2 Participants and interview findings**

A total of 70 male patients with alcohol dependence were enrolled. Participants ranged in age from 21 to 58 years. Educational levels ranged from primary school to master’s degree. Heights ranged from 160 to 192 cm, and weights from 54 to 112 kg. The cumulative duration of drinking ranged from 2 to 40 years, with daily alcohol consumption between 33.9 and 424 g.

The results showed that patients with alcohol dependence most frequently preferred drinking at home (62/124) and in restaurants (39/124). Their greatest concerns regarding the consequences of drinking were being criticized or quarreling with family members (25/154), followed by developing chronic diseases or disabilities (24/154), accidental death due to drinking (23/154), and impulsively harming family members (22/154). Nearly half of the patients reported being most unwilling to drink with family members or relatives (38/91), followed by partners (22/91). In addition, a considerable proportion indicated that they were least willing to drink when feeling happy (34/98).

Based on these findings, four aversive scenarios were developed and corresponding scripts were written.

**S1.3 Scenario scripting and construction**

The four aversive scenarios were as follows:

(1) After long-term heavy drinking, the protagonist quarrels with his wife at home, physically assaults her after she discards his alcohol, and is subsequently sent by his wife and younger brother to a psychiatric hospital for compulsory treatment.

(2) The protagonist drinks at night in snowy weather, collapses on the way home, and dies from hypothermia.

(3) The protagonist rides a bicycle home alone on a rainy night after drinking and sustains injuries in a fall.

(4) The protagonist vomits into a toilet after excessive drinking.

The first storyline depicted a man who, after long-term heavy drinking, neglected work and family responsibilities. One afternoon, upon returning home, he attempted to drink but was refused by his wife. This led to a heated argument over his persistent drinking. When his wife discarded his alcohol, he physically assaulted her. Crying, the wife called her younger brother, who rushed over, confronted the man with violence, and, after discussion with the wife, restrained him and forcibly escorted him to the addiction treatment department of a psychiatric hospital.

The second storyline showed the man drinking with friends and then walking home alone on a snowy night, still carrying and drinking from a bottle. While taking a remote path, he collapsed in the snow due to excessive alcohol intake. He was found the next day, having died of hypothermia.

The third storyline depicted the man drinking with friends and then riding an electric bicycle home alone on a rainy night. Because of heavy alcohol consumption, he was unable to maintain balance and fell. His immediate reaction after the fall was not to check for injuries but to see whether his alcohol bottle had been broken.

The fourth storyline involved the man drinking large amounts of beer at home with friends. Due to excessive intake, he rushed to the bathroom, where he vomited violently.

**S1.4 Actors, filming sites, and equipment**

Three professional actors from a film production company were selected. The leading actor, who played the role of a patient with alcohol dependence, had extensive experience in social drinking activities. Prior to filming, all three actors received training from professionals with more than 10 years of experience in the Department of Addiction Medicine. The training was designed to familiarize the actors with the clinical manifestations and psychological characteristics of patients with alcohol dependence, thereby enhancing the authenticity of the scenarios.

The first scenario was filmed in an approximately 80 m² family residence and a psychiatric hospital, realistically reproducing the appearance of a typical Chinese household and hospital environment. The second scenario was filmed on a snowy road, where the snow emphasized the cold temperature and the risk of hypothermia. The third scenario was filmed on a road at night following rainfall to evoke the impression of a slippery surface and the imminent risk of falling. The fourth scenario was filmed in the bathroom of a family residence, where the addition of vomitus provided a strong aversive visual stimulus.

Filming was conducted using the Insta360 ONE RS 1-Inch Edition panoramic camera, which was mounted on a motorcycle helmet to achieve first-person perspective recording (Figure S2). The videos were subsequently edited and processed and then played on the VIVE Focus 3 device, which features 5K resolution and supports the playback of 360° panoramic videos.

**S1.5 Preliminary validity evaluation**

We recruited 50 patients with alcohol dependence to undergo aversion therapy using the four aversive scenarios. All patients reported that the scenarios were realistic and that each of them elicited aversive responses.

**Supplementary Methods S2. Development and validation of VR cue-exposure scenarios**

**S2.1 Interview-based design rationale**

Chen et al[1]. conducted semi-structured interviews with male patients with alcohol dependence hospitalized in the Addiction Medicine Center of the Second Affiliated Hospital of Henan Medical University between March and May 2022. All participants met the ICD-10 diagnostic criteria for alcohol dependence. The study was registered in the Chinese Clinical Trial Registry (ChiCTR2200059836) on May 12, 2022, and received ethical approval from the Ethics Committee of the Second Affiliated Hospital of Henan Medical University (Approval No. XYEFYLL-(Research)-2022-32).

**S2.2 Participants and scene selection**

A total of 50 male patients with alcohol dependence were recruited for semi-structured interviews. Participants ranged in age from 21 to 57 years, with 9 to 17 years of education. The cumulative duration of alcohol consumption ranged from 1 to 41 years, the duration of dependence from 0.25 to 30 years, and daily alcohol intake from 62.4 to 624 g.

The results showed that patients with alcohol dependence most commonly consumed spirits (49/50). All patients reported drinking at home (50/50), and a proportion also reported drinking in restaurants (34/50). Solitary drinking was reported as the most frequent pattern (50/50), while many participants also reported drinking with friends in specific social contexts (30/50), reflecting context-dependent drinking behaviors rather than a contradiction. Based on these findings, the scenario of drinking alone in a restaurant was selected as the cue-exposure scenario.

**S2.3 Construction of the VR cue-exposure environment**

We collaborated with Xi’an Huiyun Medical Technology Co., Ltd. to construct the cue-exposure scenario. The scenario was developed using the Unity engine and primarily included a liquor cabinet, a cold dish counter, a refrigerator, dining tables, and two televisions, with a total area of 70 m². Participants were able to move freely within the environment, with a 360° rotatable view that allowed them to observe every angle and corner of the scene. A total of 17 alcohol-related cues and 17 non-alcohol-related cues were placed on the dining tables.

**S2.4 Recording of eye-tracking and grasping behavior**

Using motion capture technology integrated into the HTC VIVE Focus 3 VR headset, participants could reach for and grasp either alcohol-related or non-alcohol-related cue models on the tables. These actions were recorded by the alcohol craving assessment system developed by Xi’an Huiyun Medical Technology Co., Ltd., which documented variables including time to grasp alcohol-related cues, number of alcohol-related grasps, time to grasp non-alcohol-related cues, and number of non-alcohol-related grasps.

In addition, eye-tracking technology provided by the HTC VIVE Focus 3 eye-tracking accessory was used to record participants’ gaze behavior. Any point in the environment that participants fixated on was captured by the eye-tracking device. When participants gazed at alcohol-related or non-alcohol-related cues, these data were likewise recorded by the alcohol craving assessment system, including time fixating on alcohol-related cues, number of alcohol-related fixations, time fixating on non-alcohol-related cues, and number of non-alcohol-related fixations (Figure S3).

**S2.5 Preliminary validity evaluation**

We recruited 50 patients with alcohol dependence to undergo cue exposure using the virtual reality scenario. All patients reported that the scenario was realistic, and the scene elicited sensitive cue-grasping and eye-tracking responses, indicating effective cue reactivity.

**Supplementary Methods S3. Development and validation of VR relaxation scenarios**

**S3.1 Equipment**

The relaxation condition was delivered using the HTC VIVE Focus 3 head-mounted VR device. As in the other VR conditions, the relaxation scenario was presented in the form of 360° panoramic videos. Videos were recorded using an Insta360 ONE RS 1-Inch Edition panoramic camera mounted on a tripod for fixed-position filming.

**S3.2 Scene design and filming locations**

The relaxation scenario was designed to provide calming content unrelated to alcohol cues and to avoid discomfort for participants. Filming was conducted in Diqing, Yunnan, and Huixian, Xinxiang. Six relaxation scenes were ultimately selected: Roadside Viewing Platform, Rural Scenery, Grassland Yak, Canyon River, Quiet Lake Surface, and Snow Mountain in the Distance (Figure S4).

**S3.3 Preliminary validity evaluation**

After production was completed, 20 patients with alcohol dependence were invited to view the six relaxation scenarios. All participants reported that they were able to accept viewing the videos using the VR device, and all stated that each relaxation scenario induced a sense of relaxation.

**Supplementary Questionnaire 1. Semi-structured interview questionnaire for aversion scenario development**

Hello! Welcome to this study, which focuses on identifying triggers for alcohol aversion. We will keep all survey content confidential. Please answer the interview questions truthfully. Thank you very much for your cooperation.

Project Number： Name abbreviation：

**General demographic information**

1. Gender： ①male ②female
2. Age：
3. Height：
4. Weight：
5. Educational level：
6. Nation：
7. Marital status：
8. Employment situation：
9. Left or right handedness：

**Alcohol usage**

1. Accumulated years of alcohol consumption：
2. Daily alcohol consumption： （Need to indicate the alcohol content of the wine）
3. Drinking places： （Select at least one option and at most three options）

①home②restaurant③barbecue stall④employer⑤bar

1. Most afraid of the consequences of drinking alcohol： （Select at least one option and at most three options）

①Impulsiveness hurts family members②Causing chronic diseases or disabilities③Conflict with outsiders④Impulse buying⑤Crime or arrest⑥Sent to the hospital for compulsory abstinence from alcohol⑦Delay in work or dismissal⑧Experiencing discomfort such as vomiting and headache⑨Accused or argued with by family members⑩Accidental death occurred⑪Causing family breakdown

1. Who do you least want to drink with： （Select at least one option and at most three options）

①work partner②alone③friend④family or relatives⑤companion

1. The emotion of least wanting to drink： （Select at least one option and at most three options）

①fatigue②anxiety③happy④pressure⑤sadness⑥angry⑦bored⑧relax

**Supplementary Figures**

**Figure S1. Screenshots of the four aversive scenarios**


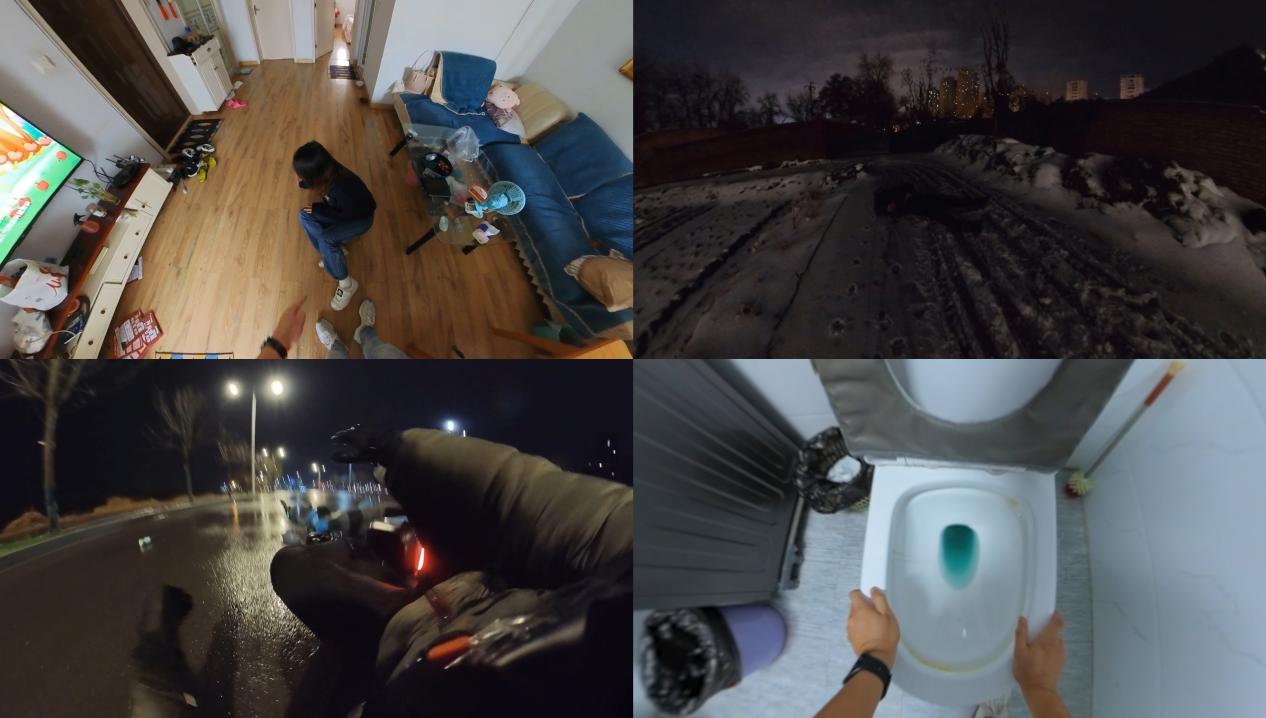


**Figure S2. Filming equipment for aversion scenarios**


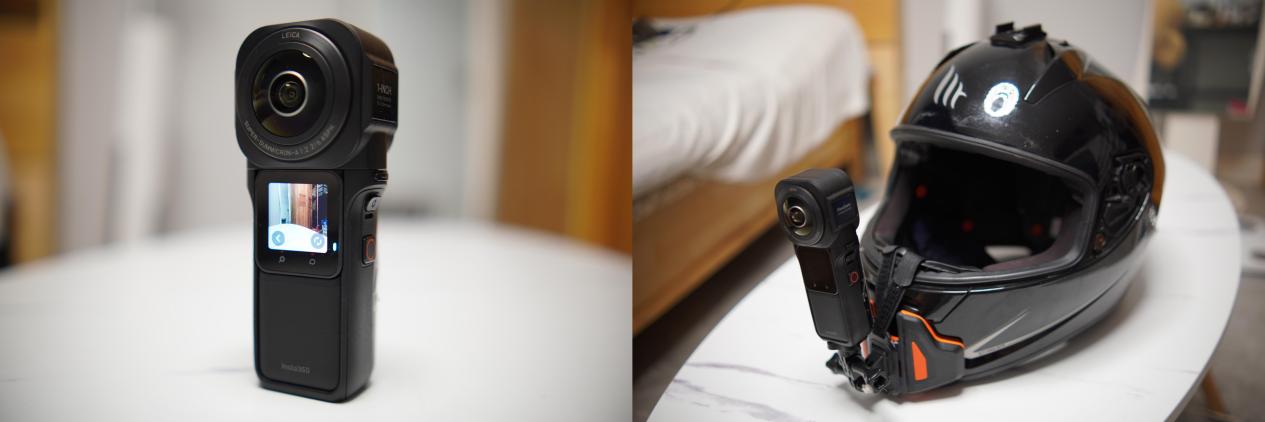


**Figure S3. Screenshot of the cue-exposure scenario**


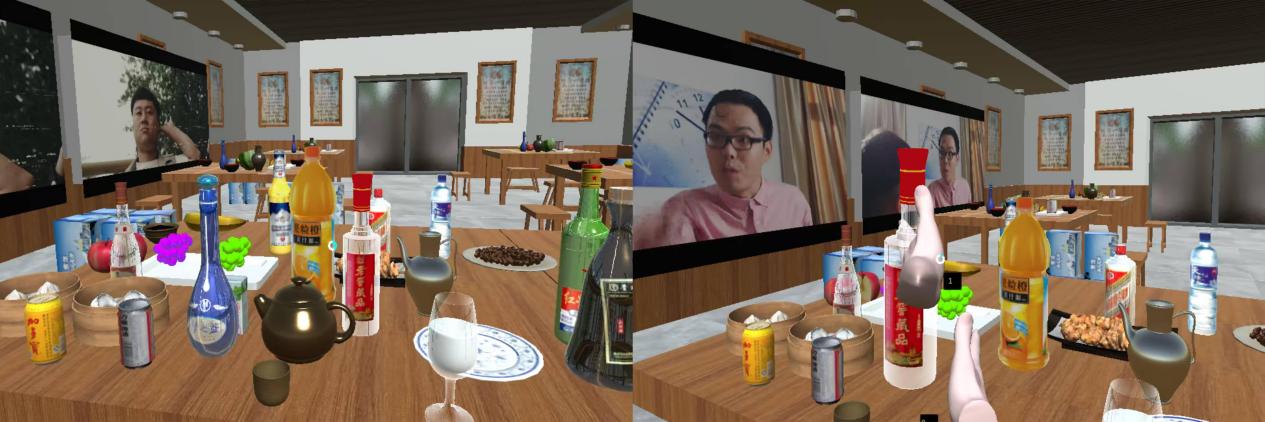


**Figure S4. Six relaxation scenarios**

**
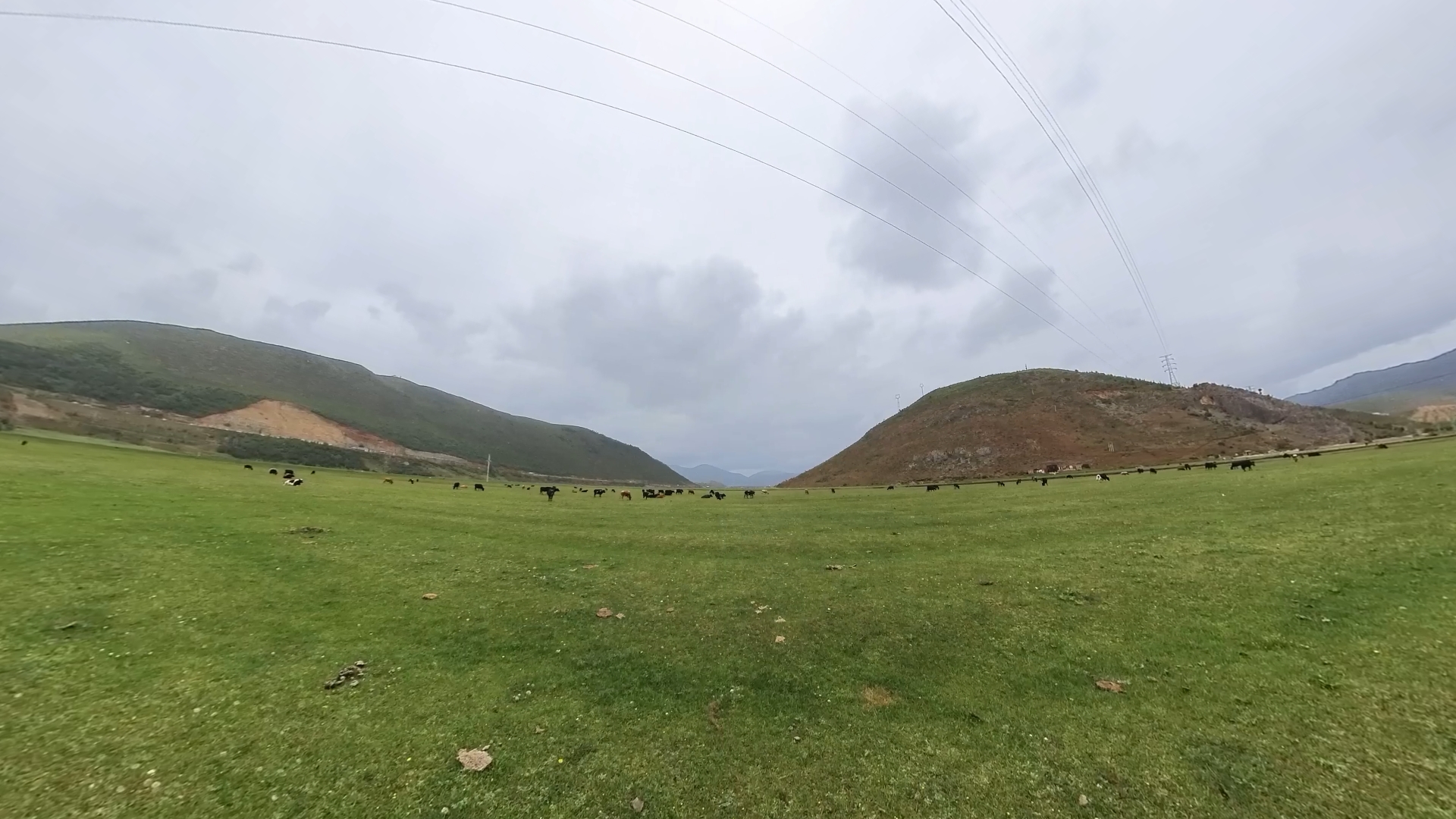

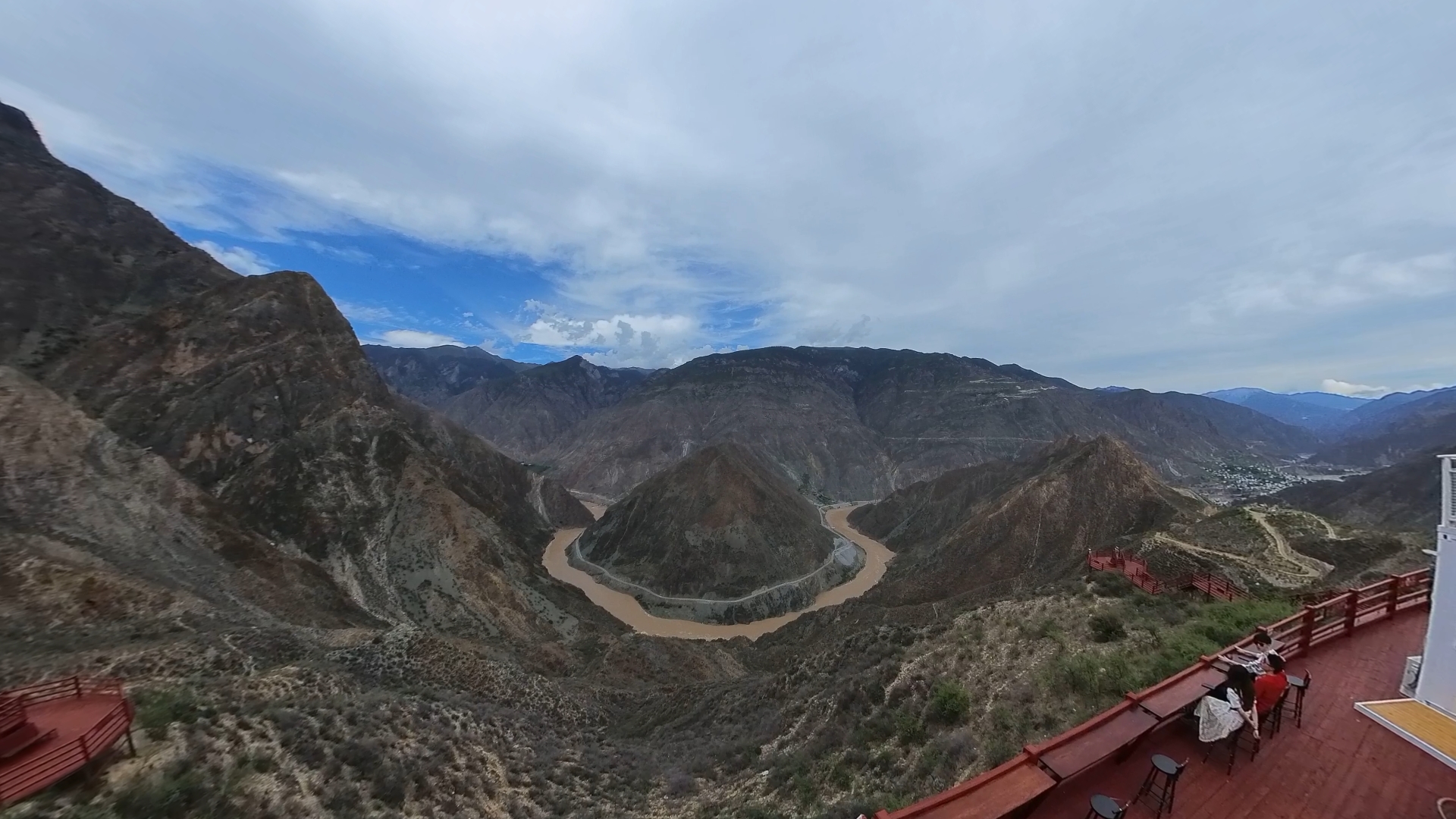

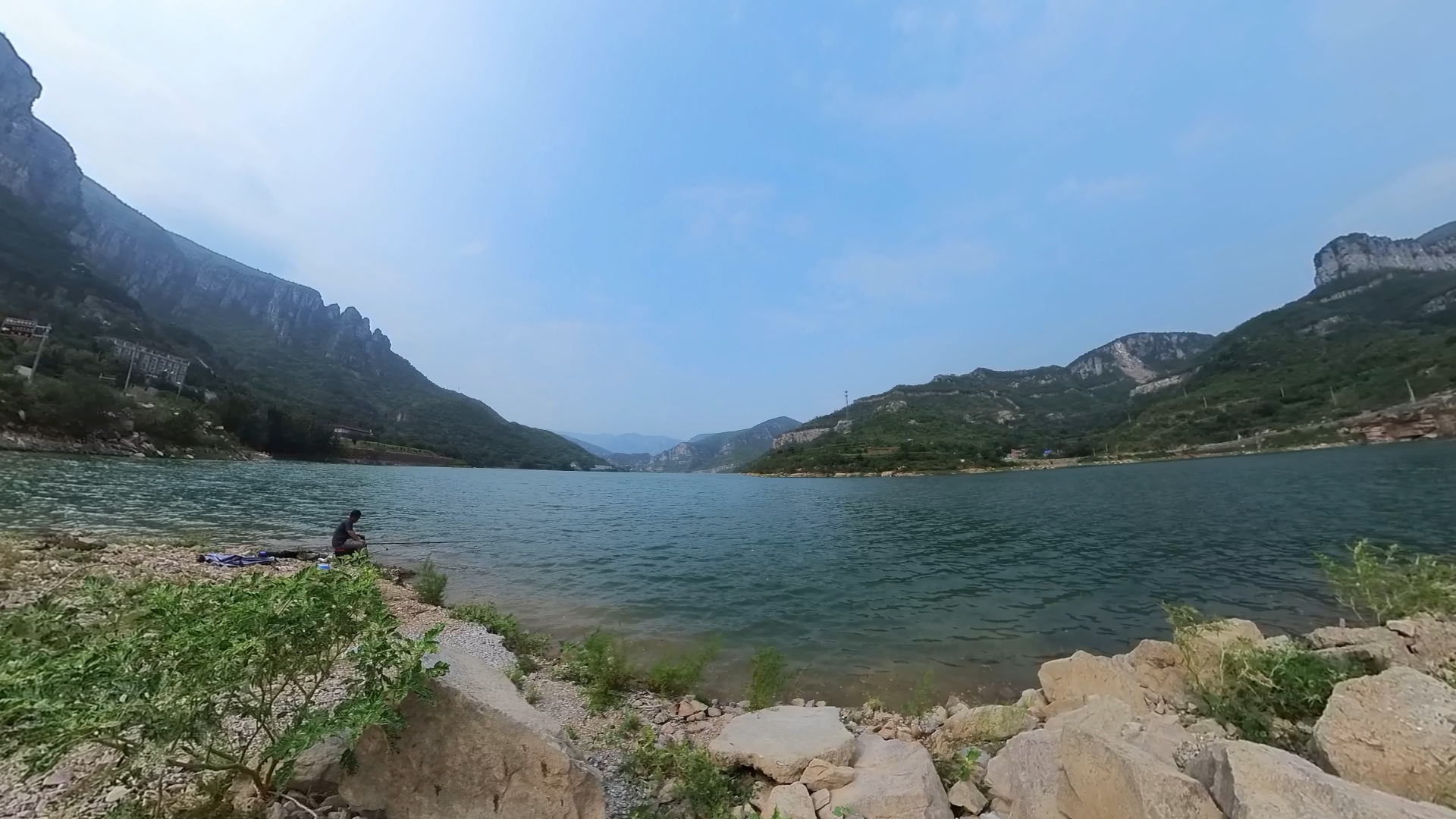

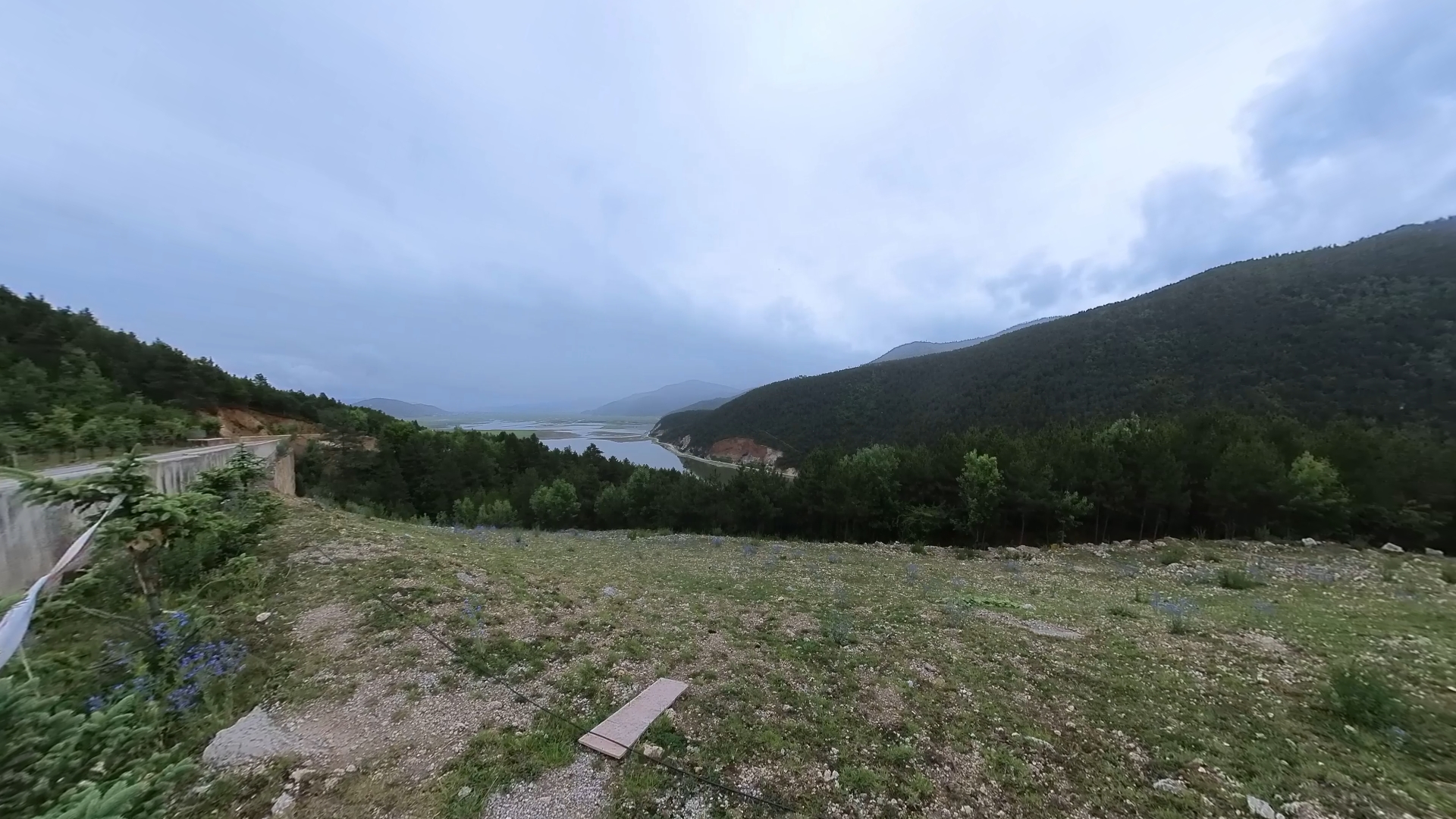

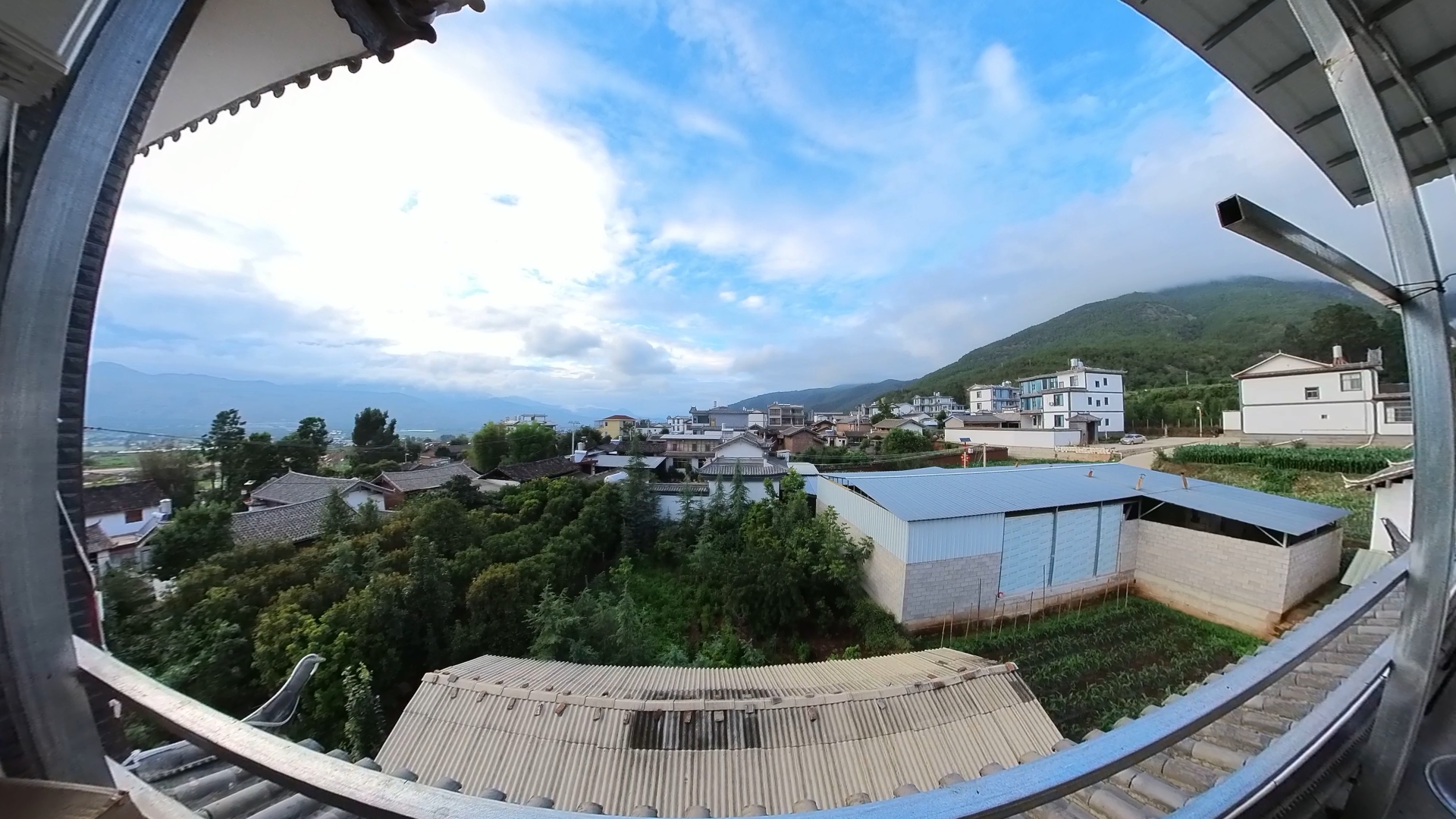

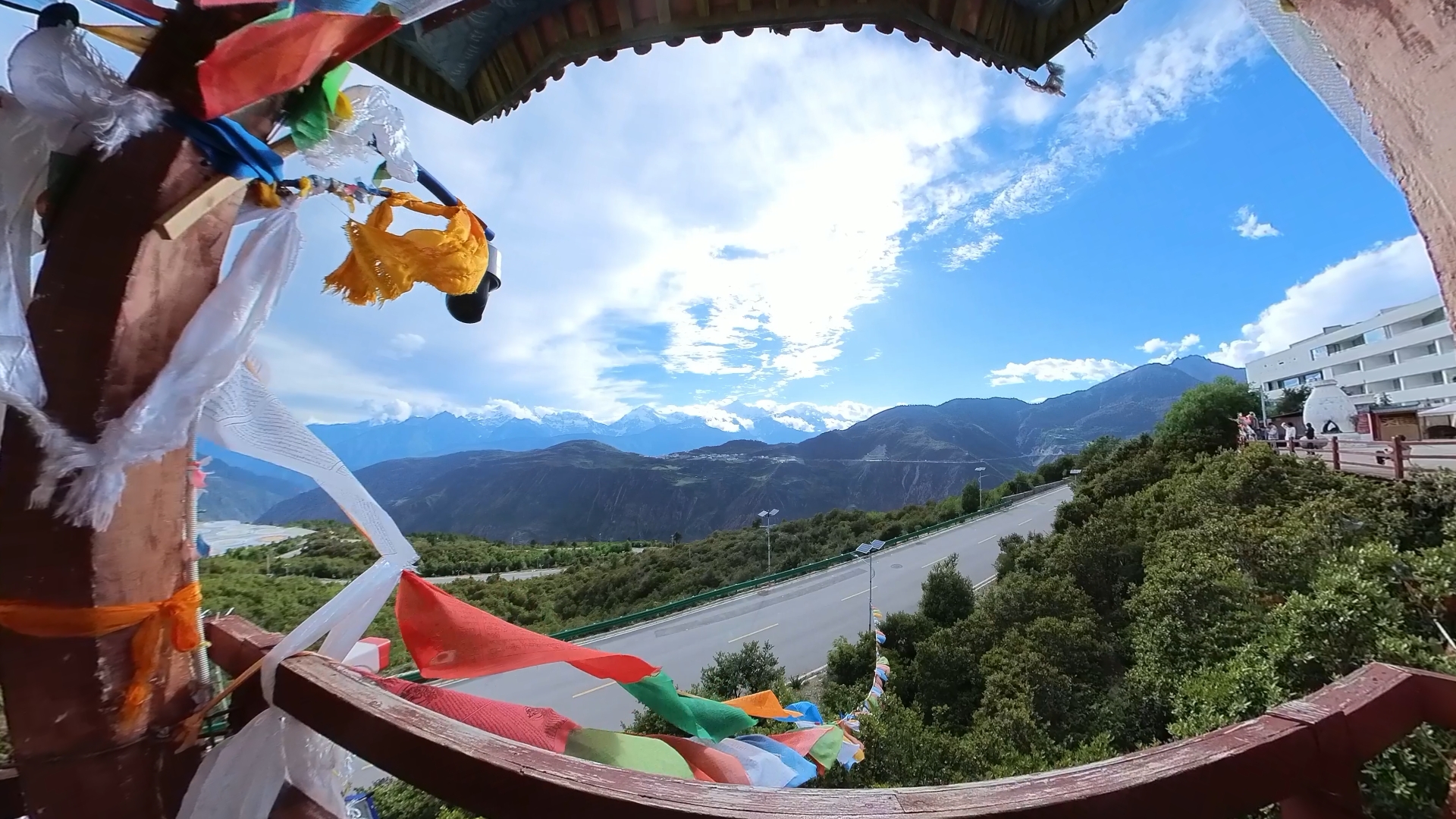
**

**References**

1. Chen Ming. Application Research of Virtual Reality Combined with Eye Tracking and Motion Capture Technology in Alcohol Dependence [D]: Henan Medical University, 2025
